# Species-specific loop-mediated isothermal amplification (LAMP) assays for the detection of three blood flukes affecting bluefin tuna aquaculture

**DOI:** 10.1007/s00436-026-08775-2

**Published:** 2026-09-30

**Authors:** Melissa J. Carabott, Cecilia Power, Sho Shirakashi, Paul A. Ramsland, Barbara F. Nowak, Nathan J. Bott

**Affiliations:** 1 Department of Biology, School of Science, RMIT University, Melbourne, VIC 3083 Australia; 2 Aquaculture Research Institute, Kindai University, Wakayama, 649-2211 Japan; 3 Department of Surgery, Austin Health, The University of Melbourne, Heidelberg, VIC 3084 Australia; 4 Institute for Marine and Antarctic Sciences (IMAS), University of Tasmania, Locked Bag 1370, Launceston, TAS 7250 Australia

**Keywords:** Aquaculture, *Cardicola* spp., Molecular diagnostics, Field-based diagnostics, Non-lethal sampling, Gill biopsy, Pacific bluefin tuna, Blood flukes

## Abstract

Blood flukes of the genus *Cardicola* (Digenea: Aporocotylidae) are well-documented health threats to farmed and ranched bluefin tuna (BFT), where infections can result in substantial economic losses. While recombinase polymerase assays (RPA) have been developed for two species of *Cardicola*, the high degree of sequence conservation across all three species reported from BFT requires an alternative method to enable confident species-level discrimination. The aims of this study were to design, optimise and validate loop-mediated isothermal amplification (LAMP) for the detection of the three *Cardicola* species which infect BFT, and apply the assays to Pacific bluefin tuna (PBT) cultured in Japan. Species-specific LAMP assays were successfully designed, optimised and validated for *C. forsteri*, *C. opisthorchis* and *C. orientalis* targeting ITS-2 ribosomal DNA. In addition, these assays were applied to DNA from three sample types targeting sites of significant pathology, the heart, gill necropsy and gill biopsy of 15 PBT. Results were compared to outputs of quantitative polymerase chain reactions (qPCR), the gold standard for blood fluke detection in ranched Australian SBT, where LAMP assays were shown to be more sensitive. This research constitutes the first development and application of species-specific LAMP diagnostics for the three described *Cardicola* species infecting BFT, providing a rapid, cost-effective and accessible alternative to standard molecular diagnostics, informing management and treatment decisions.

**Supplementary Information:**

The online version contains supplementary material available at https://doi.org/10.1007/s00436-026-08775-2.

## Introduction

*Cardicola* (Digenea: Aporocotylidae) blood flukes are well-documented parasites of bluefin tuna (*Thunnus* spp., BFT), posing a significant health risk to farmed and ranched aquaculture stocks (Ellis and Kiessling 2016). As a high-value species traded extensively on the international market, BFT health management is critical to maintaining production and profitability, where disease outbreaks can have serious financial and welfare implications (Ellis and Kiessling 2016). Across BFT species, blood flukes demonstrate a conserved infection strategy causing cardiorespiratory pathology. Adult blood flukes either inhabit the heart or the gills, with *C. forsteri* found in both sites, *C. opisthorchis* primarily in the heart and *C. orientalis* in the gills (Cribb et al. 2000; Ogawa et al. 2010; Polinski et al. 2013; Shirakashi et al. 2013; Hudson et al. 2025). Most eggs reside within the afferent filament arteries and become lodged within the vasculature associated with the gill lamellae (Colquitt et al. 2001; Ogawa et al. 2010; Palacios-Abella et al. 2015). Pathology is primarily related to the accumulation of the eggs in the gills which can lead to the increased production of gill mucus, signs of respiratory distress, lethargy and in extreme cases mortality (Munday et al. 2003; Kirchhoff et al. 2012; Shirakashi et al. 2012a). *Cardicola orientalis* may pose a greater pathological risk than other *Cardicola* spp. because it produces more eggs and, during heavy infections, adults may obstruct blood flow within the branchial arteries (Shirakashi et al. 2012a). *Cardicola* spp. infection composition varies among BFT hosts, with all three species reported to infect Pacific bluefin tuna (*Thunnus orientalis*, PBT) farmed or ranched in Japan and Mexico as well as Atlantic bluefin tuna (*Thunnus thynnus*, ABT) ranched in the Mediterranean, while *C. forsteri* and *C. orientalis* infect ranched Southern bluefin tuna (*Thunnus maccoyii*, SBT) in Australia (Cribb et al. 2000; Mladineo and Tudor 2004; Ogawa et al. 2010, 2011; Ballí Garza et al. 2016; Shirakashi et al. 2016). Current commercial control of *Cardicola* spp. relies heavily on anthelmintic praziquantel (PZQ), which has been shown to significantly reduce adult blood fluke prevalence and BFT mortalities (Hardy-Smith et al. 2012; Shirakashi et al. 2012a; Ishimaru et al. 2013; Power et al. 2019). However, PZQ is not a residual treatment and reinfections can occur, where PZQ is ineffective against eggs containing viable miracidia (Shirakashi et al. 2012a; Power et al. 2019, 2021, 2023; Carabott et al. 2023).

Traditionally, diagnosis relied on morphology, identifying *Cardicola* spp. by microscopic examination of adults isolated from hearts or gills or eggs in gill filaments, or by in situ histological sectioning of key infection sites (Aiken et al. 2006; dos Santos et al. 2012; Shirakashi et al. 2012b). While simple and easy to perform, morphological diagnostics are limited to detecting a single life cycle stage and often overlook migrating stages, where reliable species discrimination is hindered by morphological similarity (Shirakashi et al. 2012b; Polinski et al. 2013). Differentiating species using highly specific diagnostic tools is crucial to blood fluke monitoring, as the three *Cardicola* species differ in their pathogenicity and infection dynamics across BFT host species or within host organs (Aiken et al. 2007; Shirakashi et al. 2012b, 2016; Ishimaru et al. 2013; Palacios-Abella et al. 2015; Power et al. 2020). Species-specific quantitative polymerase chain reaction (qPCR) assays targeting the internal transcribed spacer-2 (ITS-2) region of ribosomal DNA (rDNA) have enabled sensitive detection and differentiation of *Cardicola* spp. at any life stage, including during low severity infections (Polinski et al. 2013; Neumann et al. 2018). Considered the gold standard for blood fluke diagnostics in SBT aquaculture, qPCR is routinely used to monitor the presence of *Cardicola* DNA in SBT throughout the harvest season (Murray and Cappello 2008; Power et al. 2019, 2021, 2023; Carabott et al. 2023). Despite their reliability, PCR-based assays are not practical for field use as they are prone to contamination, sensitive to sample nucleic acid purity and require specialised laboratory equipment, resulting in delays due to sample transport (Mori et al. 2001; Nwe et al. 2024). These limitations highlight the need for a sensitive, species-specific, rapid, cost-effective, and accessible method suitable for field-based detection and differentiation of blood fluke species. Isothermal amplification techniques, including recombinase polymerase amplification (RPA) and loop-mediated isothermal amplification (LAMP), have received considerable attention as they have overcome the limitations of PCR-based assays (Nwe et al. 2024). The reactions are rapid, sensitive and specific, and operate at a constant temperature, requiring only simple laboratory equipment such as a heat block for amplification (Mori et al. 2001). RPA assays were recently published for *C. forsteri* and *C. orientalis* (Power et al. 2025). However, assays have not been developed to target *C. opisthorchis*, a significant pathogen in PBT and ABT aquaculture (Ogawa et al. 2011; Palacios-Abella et al. 2015). Given the high sequence conservation across all three species, particularly between *C. forsteri* and *C. opisthorchis* (ITS-2 similarity: 94.52%), designing species-specific assays using the two-primer format of RPA is challenging. Therefore, an alternative method is required to support confident species-level discrimination in other BFT species.

LAMP offers a promising alternative because it uses *Bst* DNA polymerase and multiple primers to target six to eight distinct regions of the target sequence, providing high specificity and supporting confident discrimination between closely related species (Notomi et al. 2000; Nwe et al. 2024). In aquaculture, LAMP has been applied for the rapid detection of bacterial and parasitic pathogens in fish, highlighting its suitability for aquatic animal health diagnosis (Cai et al. 2010; Pradeep et al. 2016; Zan et al. 2022). The reaction can also provide visual confirmation of amplification without specialised equipment, supporting rapid detection in resource-limited settings (Notomi et al. 2000; New England Biolabs, 2023). One approach is a pH-dependent colour change, where protons released during DNA polymerisation lower the reaction pH, producing a visible colour change from pink to yellow (Aula et al. 2023; New England Biolabs, 2023). Leveraging these advantages, this study aimed to design, optimise and validate species-specific LAMP assays for the detection of the three *Cardicola* species described to infect BFT. The assays were subsequently applied to DNA extracted from heart, gill necropsy and gill biopsy samples collected from 15 PBT collected from Oshima Station, Aquaculture Research Institute operated by Kindai University, in 2024. This enabled comparison of LAMP performance with qPCR across the available pathology-relevant sample types.

## Materials and methods

### Synthetic DNA standards

Double-stranded synthetic DNA sequences (gBlock^®^; Integrated DNA Technologies, USA) were used as standards throughout LAMP validation and optimisation. Sequences corresponded to the ITS-2 region of rDNA specific to *C. forsteri*, *C. opisthorchis* and *C. orientalis*. Synthetic sequences were serially diluted to obtain standards.

### Species-specific LAMP primer design

Sequences of the ITS-2 region of rDNA were retrieved for *C. forsteri* (Accession number: EF661575.1), *C. opisthorchis* (Accession number: HQ324228.1) and *C. orientalis* (Accession number: HQ324226.1) from National Centre for Biotechnology Information (NCBI) RefSeq database (https://www.ncbi.nlm.nih.gov/). *Cardicola orientalis* LAMP primers were designed using the New England Biolabs (NEB) LAMP Primer Design Tool (https://lamp.neb.com/#!/). However, as the ITS-2 rDNA sequences of *C. forsteri* and *C. opisthorchis* share 94.52% sequence similarity, the NEB LAMP Primer Design Tool was unable to generate species-specific primers. Therefore, LAMP primers for *C. forsteri* and *C. opisthorchis* were manually designed in Geneious Prime (Biomatters Ltd., Auckland, New Zealand). ITS-2 sequences from all three species were aligned using Clustal Omega implemented in Geneious Prime to identify regions of interspecific sequence variation, which were targeted for species-specific LAMP primer design.

**Fig. 1:**
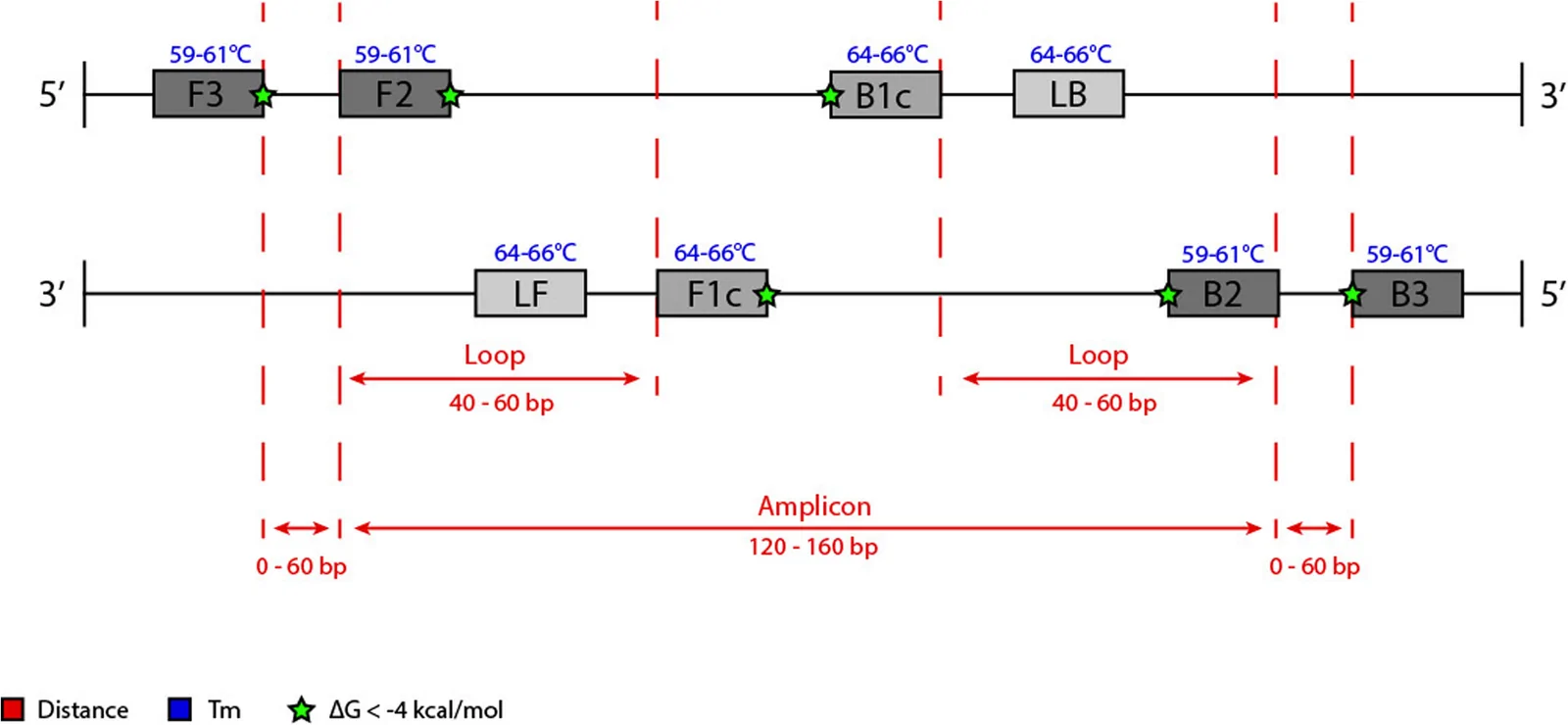
Parameters used for manual LAMP primer design, including primer arrangement, target melting temperature (Tm), spacing between primer regions and primer end stability (∆G). Outer primers = F3 and B3; inner primers = FIP (F2 and F1c) and BIP (B2 and B1c); loop primers = LF and LB. Design criteria were based on recommendations described in the PrimerExplorer V5 manual (Eiken Chemical Co., Ltd., 2019)

LAMP assays utilise four core primers [F3, B3, FIP (composed of F2 and F1c) and BIP (composed of B1c and B2)], with optional loop primers (LF and LB) that can accelerate amplification (Notomi et al. 2000; Nagamine et al. 2002). Primers were designed following established LAMP paper design guidelines, considering primer melting temperature (Tm), GC content, amplicon length, primer stability, and avoidance of secondary structures (Eiken Chemical Co., Ltd. 2019). Target Tm ranges were 64–66°C for F1c/B1c and loop primers, and 59–61°C for F2/B2 and F3/B3, with a maximum difference of 5°C targeted between primers (Fig. 1). Primer end stability was determined by the change in free energy (∆G), targeting ≤-4 at the 3’ ends of F2/B2 and F3/B3 and the 5’ end of F1c/B1c (Fig. 1). Primers were designed to minimise secondary structures by ensuring the 3’ ends were not complementary and avoiding inter-primer homology (avoiding ≥ 3 complementary bases) and homopolymer runs (avoiding ≥ 3 of the same base) within and between primers. GC content was targeted to be between 40 and 65%, with 50–60% considered preferable. The target amplicon size was 120–160 bp, with spacing between primer regions maintained according to established LAMP design recommendations (Fig. 1).

All primers were additionally screened via the National Centre for Biotechnology Information-Basic Local Alignment Sequence Tool (NCBI-BLAST) depending on their binding score to non-target species. All primers considered for *C. forsteri* and *C. opisthorchis* LAMP applications can be found in Supplementary Table S1 and S2.

### Screening potential LAMP primer sets

Due to the high similarity between *C. forsteri* and *C. opisthorchis*, initial screening of LAMP primer set was performed using the Universal WarmStart^®^ LAMP Kit (DNA and RNA) (New England Biolabs, Ipswich, MA, USA). Each LAMP reaction had a total volume of 10 µL and consisted of 9 µL reaction mix plus 1 µL of either synthetic ITS-2 gBlock DNA or DNA/RNA free water. The 9 µL reaction mix comprised of 5 µL WarmStart LAMP 2× Master Mix, 1 µL LAMP Primer Mix (10×; containing 16 µM FIP and BIP, 2 µM F3 and B3, 4 µM LF and LB), and 3 µL DNA/RNA free water. Reactions were performed in duplicate and included a positive control consisting of 1 µL of 10 pg/µL synthetic ITS-2 DNA of the target species, 1 µL of 10 pg/µL synthetic ITS-2 DNA of the closely related non-target species to assess cross reactivity and 1 µL DNA/RNA free water as a no template control (NTC). Reactions were briefly centrifuged prior to incubation at 65 °C for 30 min followed by heating at 80 °C for 5 min to inactivate *Bst* 2.0 and RTx in preparation for detection by gel electrophoresis (2% agarose) at 100 V for 40 min.

### LAMP optimisation

All further reactions were performed using the WarmStart^®^ Colorimetric LAMP Kit (DNA and RNA) (New England Biolabs, Ipswich, MA, USA) under the same reaction composition outlined in Sect.  2.3. To optimise *Cardicola* spp. assays, reaction temperature and time were adjusted independently. For temperature, reactions were incubated at 60 °C, 63 °C and 65 °C, within the recommended temperature range for *Bst* enzyme activity. The optimal temperature was then used to assess the reaction time, where samples were incubated for 10, 20 and 30 min. All optimisation reaction sets contained triplicate positive and negative controls that were terminated by heating to 80 °C for 5 min at the end of the incubation period to inactivate *Bst* 2.0 and RTx. As the positive control, 1 µL of 10 pg/µL synthetic ITS-2 DNA of the target species was used and 1 µL DNA/RNA free water was used as an NTC. A colour change from pink (negative) to yellow (positive) indicated successful DNA amplification. The result of all reaction products was confirmed by visualisation by gel electrophoresis (2% agarose) at 100 V for 60 min. The optimised reaction conditions for each species were as follows: *C. forsteri* LAMP incubation at 65 °C for 30 min; *C. opisthorchis* LAMP incubation at 60 °C for 30 min; *C. orientalis* LAMP incubation at 63 °C for 40 min with 5% v/v dimethyl sulfoxide (DMSO; Sigma-Aldrich, St. Louis, MO, USA). Optimised LAMP primers are listed in Table 1, and their binding sites are shown in Supplementary Figs. S1–S3.

**Table 1:** LAMP primers targeting ITS-2 rDNA of *Cardicola forsteri*, *C. opisthorchis* and *C. orientalis* (*LB primer sequences are identical between the *C. forsteri* and *C. opisthorchis* assays)

Name	Sequence (5’$\:\to\:$3’)
*C. forsteri* 160 bp (EF661575.1)	
F3	GCGGCTTGGAAATGGTGCTA
B3	GCAGTCGCAACTTGCAAG
FIP	ATGTTATGCATGCAATA GCATACGTGATTGCTTGCT
BIP	GCGCGCCAGTGTTAATCA CAATCCCACTCGAATACAC
LF	AACACACACATATCGAGA
LB*	CCTAATGTGACCAGTCGCA
*C. opisthorchis* 160 bp (HQ324228.1)	
F3	CGGCTTGGAAATGGTGCCA
B3	TGCAGTCGCAACTTGCAGG
FIP	TCCATATTATGCATGCAAA GCATATGTAATGGCTTGTT
BIP	TCAAGCGCGCCAGTGTTAATT CAATCCCACTCGAATACAC
LF	AATGCACACACATTTAGG
LB*	CCTAATGTGACCAGTCGCA
*C. orientalis* 150 bp (HQ324226.1)	
F3	CCTAGATGTTTACGTTTGTGTA
B3	ACAACTAGCAACCTCAGG
FIP	TCACATTAGGAAAGAGCCACATAAT GTGTTTTGCATACGTAGCAT
BIP	CGGTCGCAATCATACTCAAATTGT CTATACTAAGCCACAACCTGA
LF	TACTGGCGCGCTCAACTAATT
LB	GGAATTGTGGTAGCGGCTTGT

### LAMP specificity and sensitivity

To determine specificity, 100 pg/µL and 0.1 pg/µL of synthetic ITS-2 DNA was assessed alongside for *C. forsteri*, *C. orientalis* and *C. opisthorchis* for each respective assay. To determine sensitivity, all assays were evaluated using 10-fold serial dilutions of synthetic ITS-2 DNA prepared immediately prior to the experiment, concentrations ranged from 1 ng/µL to 1 ag/µL were evaluated (*C. forsteri* equivalent to 1,384,600,000 to 13.87 copies; *C. opisthorchis* and *C. orientalis* equivalent to 1,371,200,000 to 13.71 copies).

### PBT sample collection and processing

Sixteen PBT were opportunistically sampled between July and August 2024 from Oshima Station, Aquaculture Research Institute, Kushimoto, Wakayama Prefecture, Japan (33°27.6′ N, 135°47.5′ E) operated by Kindai University. All PBT sampled were hatchery raised and included harvested PBT (*n* = 11) as well as moribund fish (*n* = 2) and mortalities (*n* = 3).

Sample collection was conducted in accordance with the Guidelines for Animal Experimentation at the Aquaculture Research Institute, Kindai University, Japan following previously described protocols (Carabott et al. 2023, 2026a; Power et al. 2023). Within sea cages, harvested and moribund PBT were captured by divers, stunned by percussive blow to the head and killed by a spike to the brain as per standard industrial practice with the highest consideration for animal welfare (Polinski et al. 2014). Fish were then gilled, gutted and wired, where the entire process was complete in under a minute. Samples collected from PBT included gill necropsy (*n* = 16; gold standard for blood fluke detection) and heart (*n* = 15) as well as gill biopsy (*n* = 15), a potential non-lethal sampling alternative. Sample collection followed previously described protocols (Carabott et al. 2023, 2026a; Power et al. 2023). From the gills, two samples were collected from the second left anterior holobranch based on the greater likelihood of identifying infection (dos Santos et al. 2012). First, a gill biopsy was taken from distal region of the upper gill holobranch, at approximately 1 cm in length and a maximum of 5 mm deep, typically comprising an average of 5 filaments (Carabott et al. 2026a). Second, a small 1 cm^2^ section was taken was taken from the centre of the gill for the gill necropsy (Power et al. 2019). A small section (no larger than 0.5 cm^3^) was extracted near the apex of the ventricle from the heart. Whole PBT mortalities were opportunistically collected from sea cages during sampling, where they were kept on ice and transported to Shirahama Station, Aquaculture Research Institute, Kindai University, Nishimuro, Wakayama Prefecture, Japan (33°40.8′ N, 135°21.6′ E) for processing. The exact time since death was unknown, however the fish did not show any signs of decomposition and their physical condition suggested that they died very recently. Following sample collection and dissection, all tissues, including those collected from mortalities were individually fixed in 1.5 mL RNAlater^®^ (Thermo Fisher Scientific, Scoresby, VIC, Australia) and stored at 4 °C overnight before being transferred to -20 °C until DNA extraction.

Genomic DNA from ≤ 25 mg of PBT samples were extracted using the Isolate II Genomic DNA Kit (Meridian Bioscience, Cincinnati, OH, USA). The NanoDrop™ Lite Spectrometer determined all samples to have sufficient concentrations for conducting polymerase chain reactions and samples had acceptable A260:A280 where ~ 1.8 is accepted as pure (Thermo Scientific 2012) (Table 2).

**Table 2:** Summary of DNA yield and purity from pacific bluefin tuna samples measured by NanoDrop^™^ Lite Spectrometer. Mean values and ranges are provided for each metric

	*n*	Mass (mg)	DNA concentration (ng/µL)	DNA purity (A260/A280)
Harvest PBT				
Heart	11	24.12 (21.42–27.15)	311.64 (163.10-434.50)	1.95 (1.92–1.99)
Gill necropsy	11	27.26 (23.00-32.20)	294.01 (189.90-395.90)	2.10 (2.06–2.12)
Gill biopsy	11	26.99 (24.40–29.70)	609.23 (430.80-854.80)	2.10 (2.03–2.15)
Moribund PBT				
Heart	1	23.10	289.20	1.92
Gill necropsy	2	25.00 (24.60–25.40)	389.35 (242.80-535.90)	2.27 (2.13–2.40)
Gill biopsy	2	22.53 (18.70-26.35)	381.30 (304.90-457.70)	2.10 (2.08–2.12)
Mortality PBT				
Heart	3	21.10 (15.00-25.50)	354.67 (225.80-433.20)	1.98 (1.97-2.00)
Gill necropsy	3	21.63 (20.20–23.60)	125.27 (22.10-225.40)	2.02 (1.90–2.13)
Gill biopsy	3	26.43 (25.50–27.60)	234.62 (40.45–367.10)	2.18 (2.10–2.35)

### Molecular analysis of PBT samples

PBT samples were initially analysed by qPCR. Species-specific primers and probes targeting heterogenous areas of the ITS-2 region of rDNA to detect *C. forsteri*, *C. orientalis* and *C. opisthorchis* were designed in previous studies, which confirmed their specificity (Polinski et al. 2013; Neumann et al. 2018; Power et al. 2019). Assays were performed on a Rotor-Gene™ Q (Qiagen, Hilden, Germany) and reaction cycling conditions were: 95 °C for 5 min, followed by 40 cycles of 95 °C for 10 s (denaturing) and 60 °C for 25 s (annealing). Previously generated qPCR standard curves were imported and applied without modification (Carabott et al. 2026a). PBT samples were tested in duplicate, where each reaction set included triplicate 100 pg/µL double-stranded synthetic ITS-2 DNA positive control and triplicate DNA/RNA free water no template control.

Following validation and optimisation of all three species-specific *Cardicola* LAMP assays, the same DNA elutes from PBT heart, gill necropsy and gill biopsy samples analysed by qPCR were tested using LAMP. Reactions were performed using the composition described in Sect.  2.3 and the optimised conditions described in Sect.  2.4. PBT samples were tested in duplicate, where each reaction set included 1 µL of 10 ng/µL synthetic ITS-2 DNA of the target species as a positive control and 1 µL DNA/RNA free water as a no template control.

### Statistical analysis

To evaluate the diagnostic efficacy of LAMP for PBT applications, prevalence data obtained using LAMP were directly compared with qPCR results for PBT gill necropsy, gill biopsy and heart samples. Sensitivity and specificity of the LAMP assays were calculated in reference to qPCR results. Sensitivity is the proportion of infected samples correctly detected to be positive by the non-lethal sample type out of all infected samples. Specificity is the proportion of uninfected samples correctly identified by the non-lethal sample type out of all uninfected samples. Cohen’s kappa ($\:\kappa\:$) was calculated to assess the level of agreement in identifying parasite presence or absence between the LAMP and qPCR, adjusting for chance agreement. Values were categorised as either slight agreement ($\:\kappa\:$ = 0.01–0.20), fair agreement ($\:\kappa\:$ = 0.21–0.40), moderate agreement ($\:\kappa\:$ = 0.41–0.60), substantial agreement ($\:\kappa\:$ = 0.61–0.80) and almost perfect agreement ($\:\kappa\:$ = 0.81-1.00). McNemar’s Chi-squared test was used to compare the ability to classify infection status.

## Results

### *Cardicola forsteri*

**Fig. 2:**
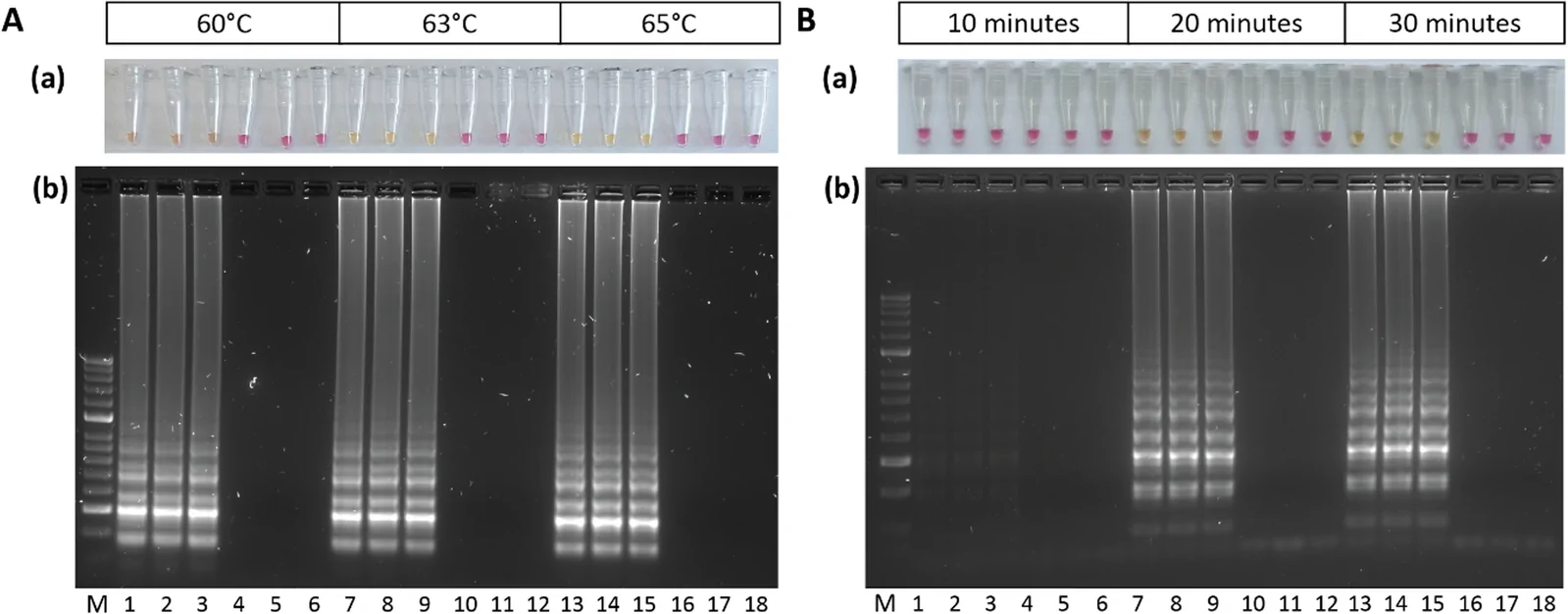
Optimisation of primer set MC1 set A to detect 10 pg/µL *Cardicola forsteri* ITS-2 DNA using LAMP based on (**a**) colorimetric visualisation followed by (**b**) 2% gel electrophoresis at 100 V for 60 min. (**A**) Varying temperatures at 60 °C, 63 °C and 65 °C for 30 min followed by a stop incubation at 80 °C for 5 min. (**B**) Varying incubation times at 10, 20 and 30 min at a constant temperature of 65 °C followed by a stop incubation at 80 °C for 5 min. For both panels, lane M. 50 bp HyperLadder; lanes 1–3, 7–9, and 13–15. 10 pg/µL *C. forsteri* ITS-2 DNA; lanes 4–6, 10–12, and 16–18. NTC

**Fig. 3:**
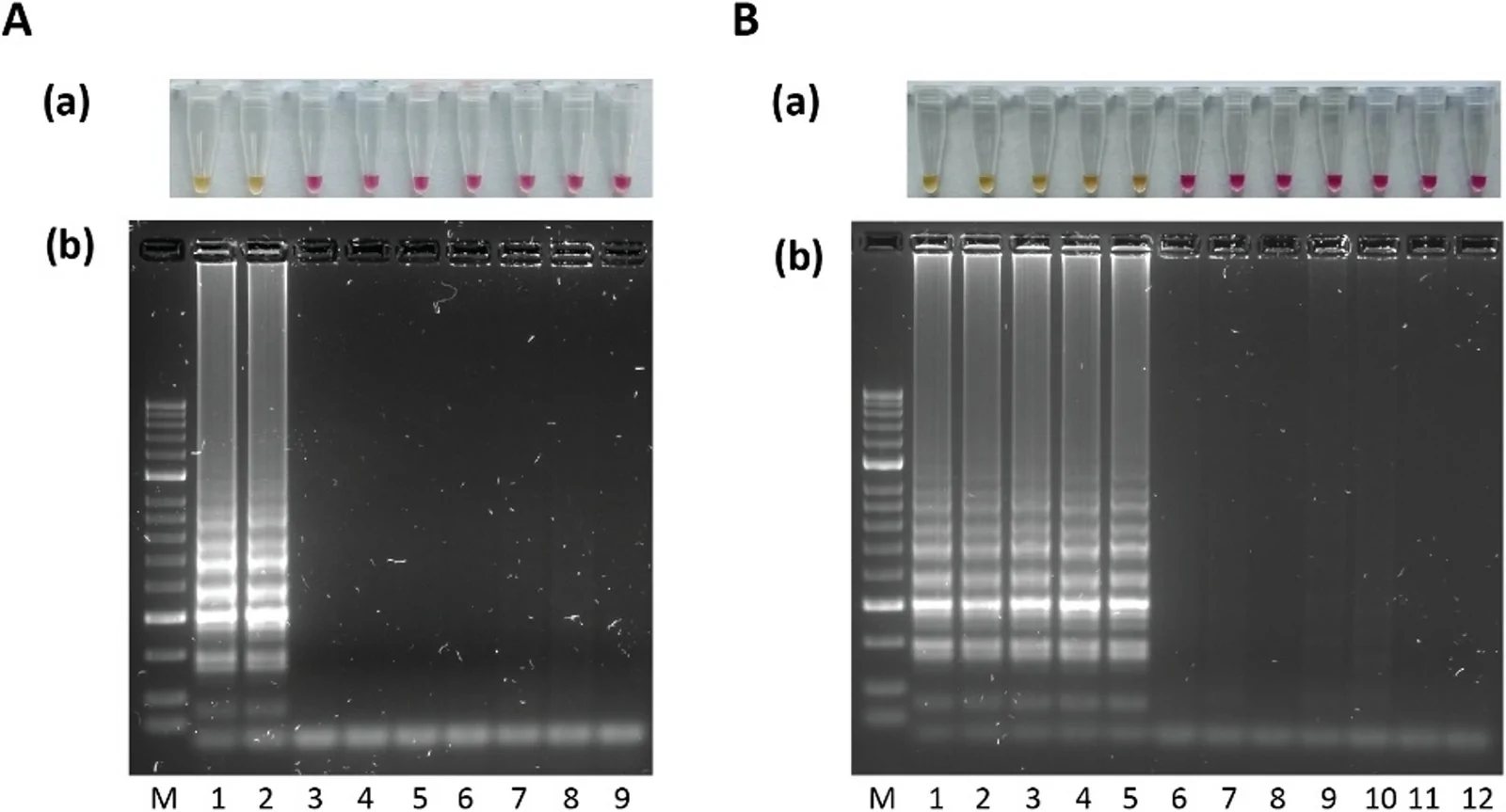
Validation of primer set MC1 set A to detect *Cardicola forsteri* ITS-2 DNA using LAMP based on (**a**) colorimetric visualisation followed by (**b**) 2% gel electrophoresis at 100 V for 60 min. (**A**) Specificity test against *C. opisthorchis* and *C. orientalis*. Lane M. 50 bp HyperLadder; lane (1) 100 pg/µL *C. forsteri* ITS-2 DNA; lane (2) 0.1 pg/µL *C. forsteri* ITS-2 DNA; lane (3) 100 pg/µL *C. opisthorchis* ITS-2 DNA; lane (4) 0.1 pg/µL *C. opisthorchis* ITS-2 DNA; lane (5) 100 pg/µL *C. orientalis* ITS-2 DNA; lane (6) 0.1 pg/µL *C. orientalis* ITS-2 DNA; lane 7–9. NTC. (**B**) Sensitivity test of *C. forsteri* DNA using serial dilutions from synthetic ITS-2 DNA. Lane M. 50 bp HyperLadder; lane (1) 100 pg/µL; lane (2) 10 pg/µL; lane (3) 1 pg/µL; lane (4) 0.1 pg/µL; lane (5) 10 fg/µL; lane (6) 1 fg/µL; lane (7) 0.1 fg/µL; lane (8) 10 ag/µL; lane (9) 1 ag/µL; lane 10–12. NTC

After initial screening of 8 potential primer sets, one was selected based on its ability to detect *C. forsteri* (Table 1; product size 160 bp) without cross-species amplification of the closely related *C. opisthorchis* (see Supplementary Table S1 and Figs. S1, S4 and S5). Lower temperatures (60–63 °C) produced strong bands; however colorimetric visualisation was more distinct at 65 °C (Fig. 2A). Incubation for 10 min did not produce an amplified product while at 20 min weak amplification in both colorimetric and gel visualisation was observed (Fig. 2B). However, incubation for 30 min resulted in a more intense colour development (Fig. 2B). Primer specificity for *C. forsteri* detection without cross-species amplification of *C. opisthorchis* and *C. orientalis* was confirmed (Fig. 3A). The assay was sensitive to 10 fg/µL (equivalent to 138,460 copies) producing bright colorimetric results with strong bands, below this concentration, amplification was not observed (Fig. 3B). Therefore, the optimal reaction conditions for *C. forsteri* LAMP amplification were 65 °C for 30 min.

### *Cardicola opisthorchis*

**Fig. 4:**
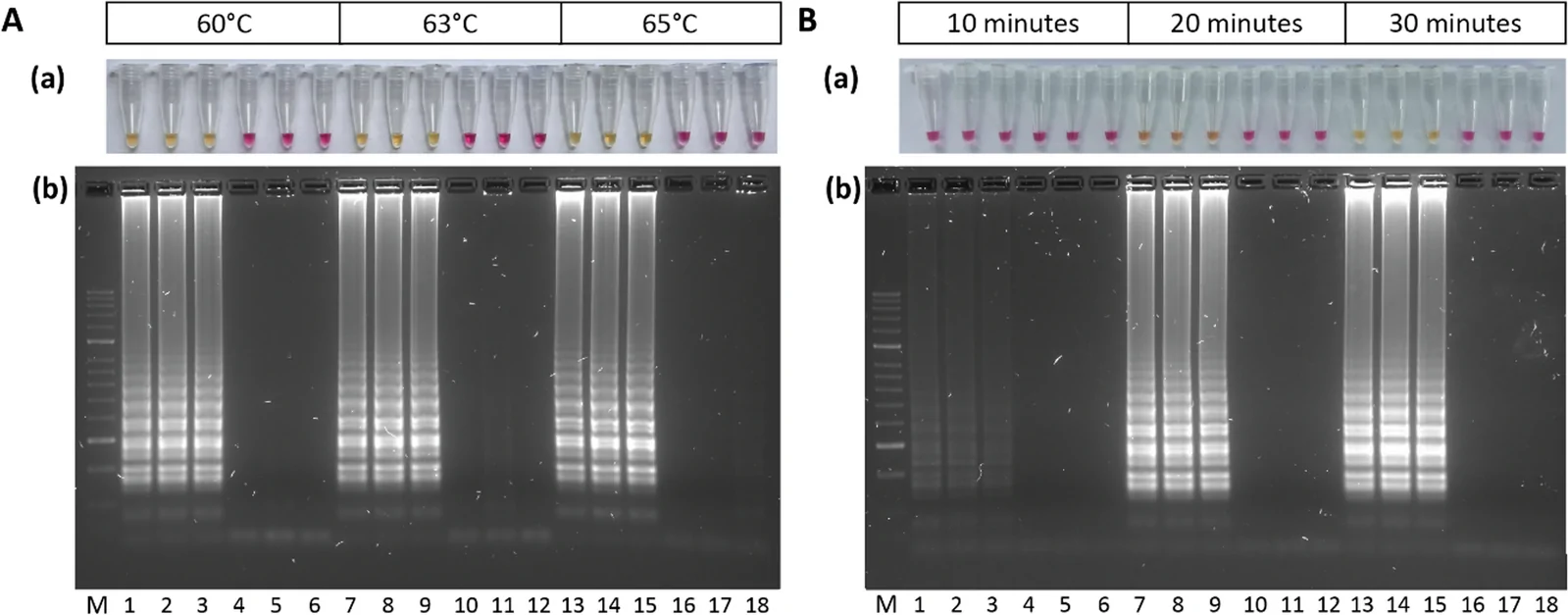
Optimisation of primer set MC1 set D to detect 10 pg/µL *Cardicola opisthorchis* ITS-2 DNA using LAMP based on (**a**) colorimetric visualisation followed by (**b**) 2% gel electrophoresis at 100 V for 60 min. (**A**) Varying temperatures at 60 °C, 63 °C and 65 °C for 30 min followed by a stop incubation at 80 °C for 5 min. (**B**) Varying incubation times at 10, 20 and 30 min at a constant temperature of 60 °C followed by a stop incubation at 80 °C for 5 min. For both panels, lane M. 50 bp HyperLadder; lanes 1–3, 7–9, and 13–15. 10 pg/µL *C. opisthorchis* ITS-2 DNA; lanes 4–6, 10–12, and 16–18. NTC

**Fig. 5:**
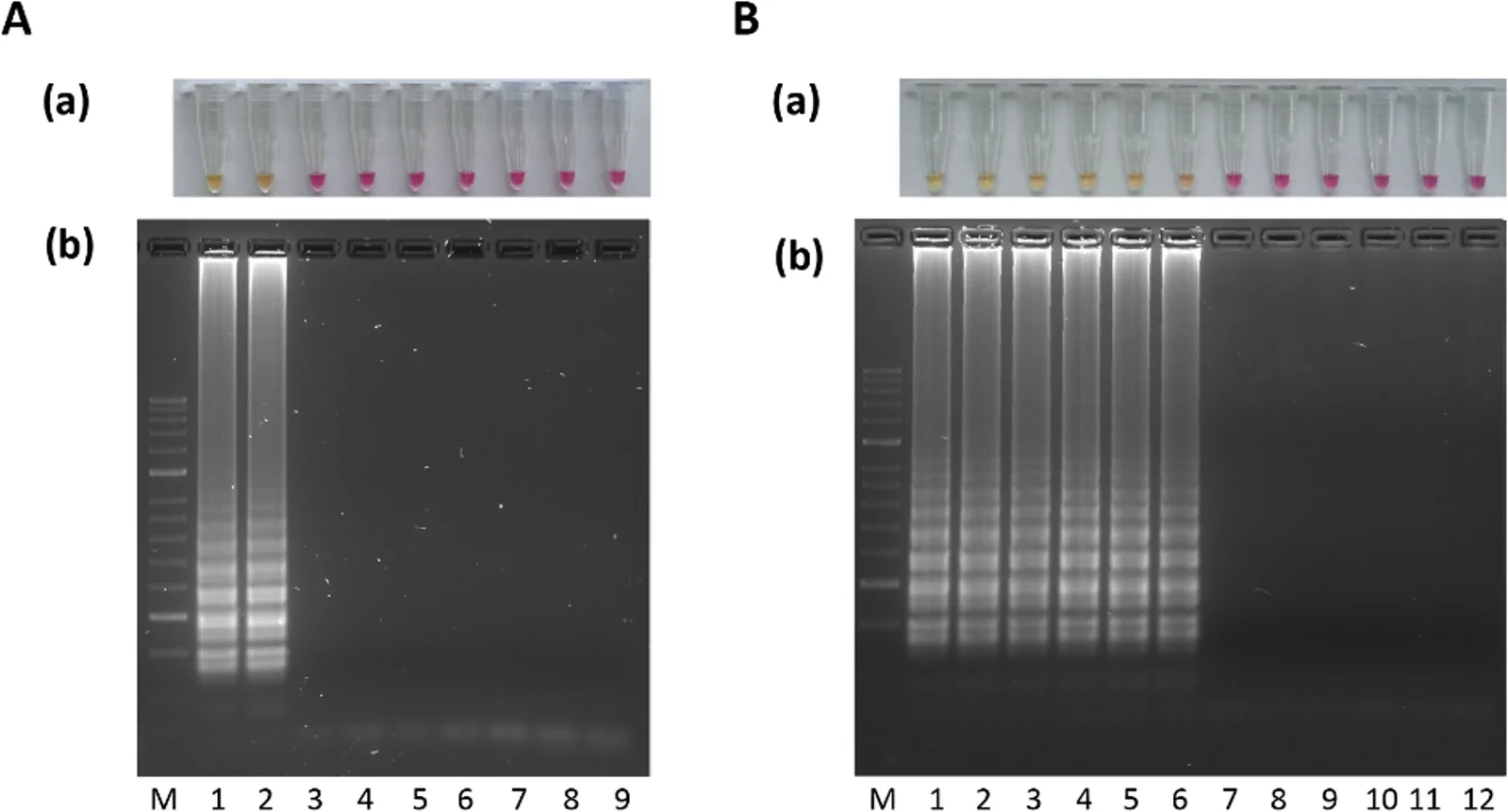
Validation of primer set MC1 set D to detect *Cardicola opisthorchis* ITS-2 DNA using LAMP based on (**a**) colorimetric visualisation followed by (**b**) 2% gel electrophoresis at 100 V for 60 min. (**A**) Specificity test against *C. forsteri* and *C. orientalis*. Lane M. 50 bp HyperLadder; lane (1) 100 pg/µL *C. opisthorchis* ITS-2 DNA; lane (2) 0.1 pg/µL *C. opisthorchis* ITS-2 DNA; lane (3) 100 pg/µL *C. forsteri* ITS-2 DNA; lane (4) 0.1 pg/µL *C. forsteri* ITS-2 DNA; lane (5) 100 pg/µL *C. orientalis* ITS-2 DNA; lane (6) 0.1 pg/µL *C. orientalis* ITS-2 DNA; lane 7–9. NTC. (**B**) Sensitivity test of *C. opisthorchis* DNA using serial dilutions from synthetic ITS-2 DNA. Lane M. 50 bp HyperLadder; lane (1) 100 pg/µL; lane (2) 10 pg/µL; lane (3) 1 pg/µL; lane (4) 0.1 pg/µL; lane (5) 10 fg/µL; lane (6) 1 fg/µL; lane (7) 0.1 fg/µL; lane (8) 10 ag/µL; lane (9) 1 ag/µL; lane 10–12. NTC

After initial screening of 8 potential primer sets, one set was selected based on its ability to detect *C. opisthorchis* (Table 1; product size 160 bp) without cross-species amplification of the closely related *C. forsteri* (see Supplementary Table S2 and Figs. S2 and S6). Clear distinct colorimetric results and strong bands were detected across all temperatures tested with the earliest bright colour change at 60 °C (Fig. 4A). Incubation for 10 min was observed to result in minimal colour change, with clear amplification detected at 20 min by both colorimetric and gel visualisation (Fig. 4B). However, a 30-minute incubation was selected as it consistently produced more intense colour development (Fig. 4B). Primer specificity for *C. opisthorchis* detection without cross-species amplification of *C. forsteri* and *C. orientalis* was confirmed (Fig. 5A). The assay was sensitive to 1 fg/µL (equivalent to 13,712 copies), producing bright colorimetric results with strong bands, below this concentration, amplification was not observed (Fig. 5B). Therefore, the optimal reaction conditions for *C. opisthorchis* LAMP amplification were 60 °C for 30 min.

### *Cardicola orientalis*

**Fig. 6:**
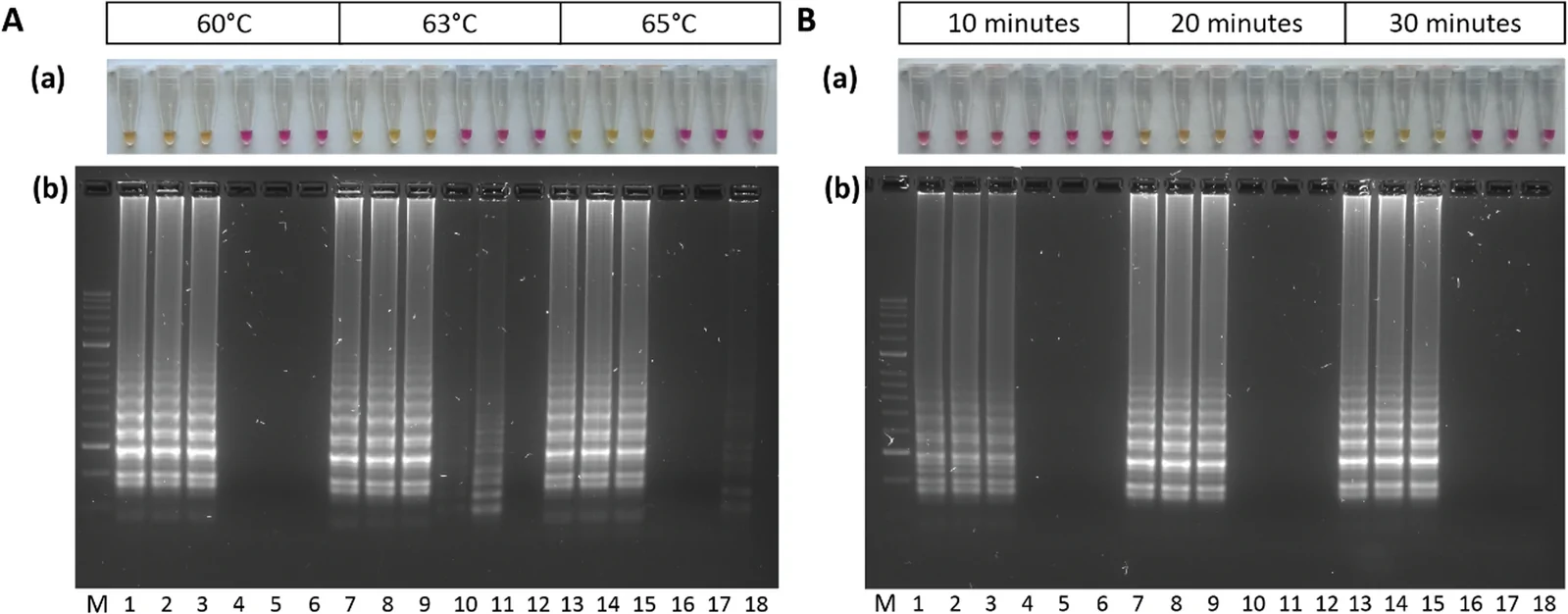
Optimisation of primer set NEB P66L39 to detect 10 pg/µL *Cardicola orientalis* ITS-2 DNA using LAMP based on (**a**) colorimetric visualisation followed by (**b**) 2% gel electrophoresis at 100 V for 60 min. (**A**) Varying temperatures at 60 °C, 63 °C and 65 °C for 30 min followed by a stop incubation at 80 °C for 5 min. (**B**) Varying incubation times at 10, 20 and 30 min at a constant temperature of 63 °C followed by a stop incubation at 80 °C for 5 min. For both panels, lane M. 50 bp HyperLadder; lanes 1–3, 7–9, and 13–15. 10 pg/µL *C. orientalis* ITS-2 DNA; lanes 4–6, 10–12, and 16–18. NTC

**Fig. 7:**
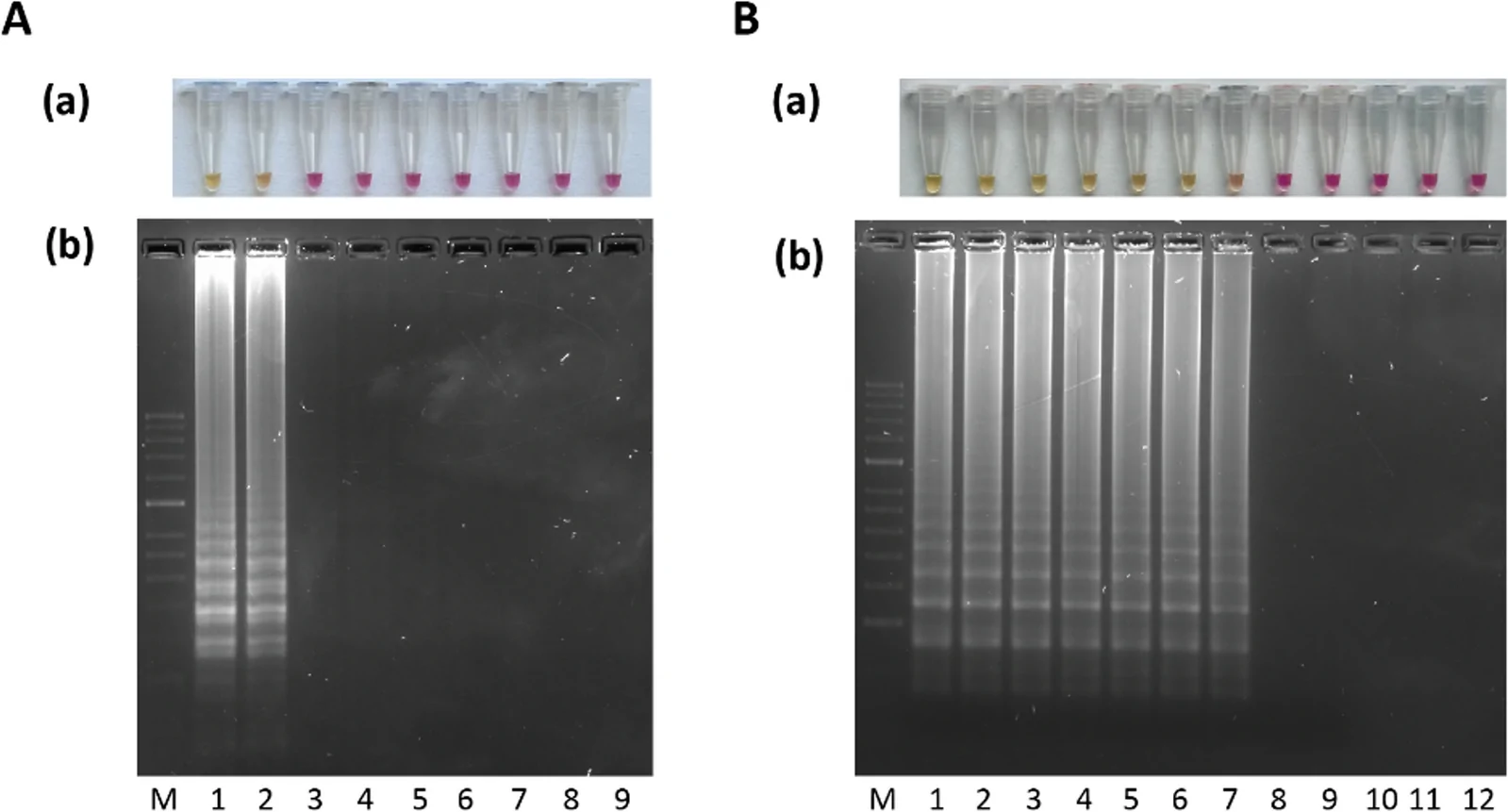
Validation of primer set NEB P66L39 to detect *Cardicola orientalis* ITS-2 DNA using LAMP with 5% v/v DMSO based on (**a**) colorimetric visualisation followed by (**b**) 2% gel electrophoresis at 100 V for 60 min. (**A**) Specificity test against *C. forsteri* and *C. opisthorchis*. Lane M. 50 bp HyperLadder; Lane (1) 100 pg/µL *C. orientalis* ITS-2 DNA; lane (2) 0.1 pg/µL *C. orientalis* ITS-2 DNA; lane (3) 100 pg/µL *C. forsteri* ITS-2 DNA; lane (4) 0.1 pg/µL *C. forsteri* ITS-2 DNA; lane (5) 100 pg/µL *C. opisthorchis* ITS-2 DNA; Lane (6) 0.1 pg/µL *C. opisthorchis* ITS-2 DNA; lane 7–9. NTC. (**B**) Sensitivity test of *C. orientalis* DNA using serial dilutions from synthetic ITS-2 DNA. Lane M. 50 bp HyperLadder; lane (1) 100 pg/µL; lane (2) 10 pg/µL; lane (3) 1 pg/µL; lane (4) 0.1 pg/µL; lane (5) 10 fg/µL; lane (6) 1 fg/µL; lane (7) 0.1 fg/µL; lane (8) 10 ag/µL; lane (9) 1 ag/µL; lane 10–12. NTC

Clear and distinct colorimetric results were observed across all temperatures tested, with 63 °C representing the lowest temperature at which the most intense yellow colour change was achieved (Fig. 6A; Table 1; product size 150 bp). Incubation for 10 min resulted in minimal colour change, with clear amplification detected at 20 min by both colorimetric and gel visualisation (Fig. 6B). However, a 30-minute incubation was selected as it consistently produced more intense colour development (Fig. 6B). Minor non-specific amplification was observed during initial optimisation when results were assessed by gel electrophoresis (Fig. 6A). Therefore, 5% v/v DMSO was incorporated into the reaction, extending the duration of the assay to 40 min to ensure clear and interpretable infection confirmation. Primer specificity for *C. orientalis* detection without cross-species amplification of *C. forsteri* and *C. opisthorchis* was confirmed (Fig. 7A). The assay was sensitive to 0.1 fg/µL (equivalent to 1,371 copies), producing bright colorimetric results with strong bands, below this concentration, amplification was not observed (Fig. 7B). Therefore, the optimal reaction conditions for *C. orientalis* LAMP amplification were 63 °C for 40 min with 5% v/v DMSO.

### Application to PBT

The developed LAMP assays for *C. forsteri*, *C. opisthorchis* and *C. orientalis* were successfully applied to DNA extracted from three sample types of 15 PBT (Supplementary Figs. S7–S9). *Cardicola* spp. infection prevalence results were compared between the developed LAMP assays and the gold standard method for detection, qPCR (Table 3). Considering all sample types, LAMP identified 12 additional positive samples for *C. forsteri*, representing a 66.67% increase in detection, where the assay failed to detect infection in one of the 18 qPCR positive samples. For *C. opisthorchis*, LAMP identified infection in 7 additional positive samples, representing a 29.17% increase in detection, where the assay failed to detect infection in one of the 24 qPCR positive samples. For *C. orientalis*, LAMP identified no additional positive samples, where the assay failed to detect infection in two of the 8 qPCR positive samples.

For the detection of *C. forsteri* ITS-2 DNA, LAMP demonstrated perfect sensitivity for gill necropsy (sensitivity = 100%) and gill biopsy samples (sensitivity = 100%) when compared to qPCR outputs, indicating LAMP detected all fish identified as infected by qPCR in either sample type. For both sample types, LAMP specificity was low, ranging from 40 to 42%), indicating a higher number of positive detections by LAMP relative to qPCR. Cohen’s kappa indicated fair agreement between the methods for gill necropsy ($\:\kappa\:$ = 0.33) and gill biopsy samples ($\:\kappa\:$ = 0.33), reflecting limited diagnostic agreement. This was supported by significant McNemar’s tests for both sample types (*p* = 0.01), which indicated preferential detection by one of the diagnostic methods, where LAMP detected infection more frequently in gill necropsy and gill biopsy samples than qPCR. In heart samples, LAMP sensitivity was also high (sensitivity = 88%), indicating LAMP to detect most fish identified as infected by qPCR. LAMP had perfect specificity (specificity = 100%), indicating perfect agreement between the diagnostic methods for identifying *C. forsteri* uninfected hearts. Cohen’s kappa indicated almost perfect agreement between the methods ($\:\kappa\:$ = 0.87), suggesting LAMP and qPCR to be effectively interchangeable in PBT hearts. This was supported by a non-significant McNemar’s test (*p* = 0.32), reinforcing the agreement between methods.

For the detection of *C. opisthorchis* ITS-2 DNA, LAMP demonstrated perfect sensitivity for gill necropsy (sensitivity = 100%) and gill biopsy samples (sensitivity = 100%) compared to qPCR outputs, indicating LAMP to detect all fish identified as infected by qPCR in either sample type. Specificity ranged from 55 to 71%, indicating that while most uninfected fish were correctly classified, there were more *C. opisthorchis* positive infections detected by LAMP relative to qPCR. Cohen’s kappa indicated substantial agreement for gill necropsy samples ($\:\kappa\:$ = 0.74) and moderate agreement for gill biopsy samples ($\:\kappa\:$ = 0.43), suggesting LAMP and qPCR to have variable results but generally good concordance depending on the sample type. This observation was supported by McNemar’s test. Gill necropsy samples had a significant McNemar’s test outcome (*p* = 0.03), indicating preferential detection by one of the diagnostic methods, where LAMP detected infection more frequently in gill necropsy and gill biopsy samples than qPCR. While McNemar’s test was not significant for gill biopsy samples (*p* = 0.16), reinforcing the agreement between methods. In heart samples, LAMP sensitivity was also high (sensitivity = 90%), indicating LAMP to detect most fish identified as infected by qPCR. LAMP also had high specificity (specificity = 80%), indicating agreement between the diagnostic methods for identifying *C. opisthorchis* uninfected hearts. Cohen’s kappa indicated substantial agreement between the methods ($\:\kappa\:$ = 0.70). This was supported by a non-significant McNemar’s test result (*p* = 0.32), reinforcing the agreement between methods.

For the detection of *C. orientalis* ITS-2 DNA, LAMP demonstrated high sensitivity for gill necropsy samples (sensitivity = 83%), indicating LAMP to detect most fish identified as infected by qPCR. LAMP also had high specificity (specificity = 80%), indicating agreement between the diagnostic methods for identifying *C. orientalis* uninfected gill necropsy samples. In contrast, gill biopsy samples had a lower sensitivity (sensitivity = 50%), indicating LAMP to detect half of the fish identified as infected by qPCR. However, LAMP had perfect specificity (specificity = 100%), indicating perfect agreement between the diagnostic methods for identifying *C. orientalis* uninfected gill biopsy samples. Although sensitivity and specificity reflected differences in detection performance between gill sample types, overall agreement between LAMP and qPCR outcomes were similar. Cohen’s kappa indicated substantial agreement between the methods for both gill necropsy ($\:\kappa\:$ = 0.61) and gill biopsy ($\:\kappa\:$ = 0.64). This was supported McNemar’s test, which was not significant for both gill necropsy (*p* = 0.56) and gill biopsy (*p* = 0.32), reinforcing the agreement between methods. In heart samples, LAMP sensitivity could not be calculated as the assay successfully identified all PBT hearts to not be infected with *C. orientalis*. Therefore, LAMP had perfect specificity (specificity = 100%), indicating perfect agreement between the diagnostic methods for identifying *C. orientalis* uninfected hearts. As no positive detections were observed for PBT hearts for either diagnostic method, Cohen’s kappa and McNemar’s test were not applicable.

**Table 3:** Comparison of LAMP and qPCR assays to detect the ITS-2 region of three *Cardicola* spp. in Pacific bluefin tuna samples. Diagnostic agreement was assessed using contingency tables (true positive (TP) – samples positive for both LAMP and qPCR; false positive (FP) – samples positive by LAMP but negative by qPCR; false negative (FN) – samples negative by LAMP but positive by qPCR; true negative (TN) – samples negative for both LAMP and qPCR)

Species	TP	FP	FN	TN	Sensitivity (%)	Specificity (%)	Kappa	McNemar
*C. forsteri*								
Gill necropsy	6	6	0	4	100	40	0.33	0.01
Gill biopsy	4	7	0	5	100	42	0.26	0.01
Heart	7	0	1	7	88	100	0.87	0.32
*C. opisthorchis*								
Gill necropsy	9	2	0	5	100	71	0.74	0.16
Gill biopsy	5	5	0	6	100	55	0.43	0.03
Heart	9	1	1	4	90	80	0.70	1.00
*C. orientalis*								
Gill necropsy	5	2	1	8	83	80	0.61	0.56
Gill biopsy	1	0	1	14	50	100	0.64	0.32
Heart	0	0	0	15	NA	100	NA	NA

## Discussion

LAMP assays were developed to detect the ITS-2 rDNA region of three *Cardicola* species reported to infect BFT, *C. forsteri*, *C. opisthorchis* and *C. orientalis*. ITS-2 rDNA was selected as it is a well-established marker for trematode diagnostics (Blasco-Costa et al. 2016; Yong et al. 2021; Mulyani and Rosidah 2022) and *Cardicola* assays have been successfully designed and applied to target the region for qPCR and RPA (Polinski et al. 2013; Power et al. 2025). Species-specific primer design had limited latitude along the region as the three *Cardicola* species had pairwise identity of 78% or greater, and similarity between *C. forsteri* and *C. opisthorchis* was greater than 94%. Although the conserved target region enabled qPCR and RPA with only two primers, designing eight compatible primers for LAMP within the highly conserved locus was inherently difficult, particularly where online primer design tools do not account for specificity. Despite this, species-specific primer sets were manually designed and demonstrated by this study to permit reliable discrimination of *C. forsteri* and *C. opisthorchis.* While a general *Cardicola* spp. assay would detect overall infection burden, they lack the capability to detect shifts in species composition. This is particularly critical for monitoring infection dynamics in response to PZQ treatment. Under repeated exposure, reductions in PZQ efficacy may emerge in a species-specific manner, where continued efficacy may be sensitive to mutations in response to PZQ with *Cardicola* spp. potentially developing at different rates (Park et al. 2021; Norbury et al. 2022). Given the reliance on PZQ as the primary control measure for *Cardicola* spp. infections in BFT, monitoring species-level dynamics is necessary to accurately assess treatment efficacy and detect early changes indicative of emerging resistance (Hardy-Smith et al. 2012).

Detection of synthetic ITS-2 rDNA standards ranged from 1,371 gene copy equivalents for *C. orientalis* to 138,460 gene copy equivalents for *C. forsteri*, which is higher than qPCR (1–5 copies) and RPA (30–50 copies) (Polinski et al. 2013; Power et al. 2025). However, when applied to DNA extracted from PBT heart and gill tissues, LAMP detected more naturally *Cardicola* infected PBT samples than qPCR. Similar discrepancies in assay performance have been reported in other helminth and fish diagnostic LAMP applications (Poon et al. 2006; Cevallos et al. 2017). This is often attributed to the susceptibility of Taq-derived DNA polymerase used in PCR to inhibitors within biological substances, even following DNA extraction (Poon et al. 2006; Cai et al. 2010; Cevallos et al. 2017). In contrast, the strand displacing *Bst* polymerase used in LAMP has a greater tolerance to sample-derived inhibitors (Notomi et al. 2000; Cai et al. 2010; Cevallos et al. 2017; Papadakis et al. 2022). From host-derived extracts, DNA of the parasite is present at low concentrations relative to host genomic material, therefore this increased tolerance to inhibitors may support improved detection. However, LAMP has also been shown to maintain detection in the presence of substantial background DNA compared to PCR, with a detection threshold 10-fold lower than PCR under comparable conditions. Another consideration may be the binding sites of LAMP primers, which target a different region on the ITS-2 than qPCR and RPA. Future work could assess the impact of the differing primer binding sites by using the LAMP outer primers (F3/B3) in qPCR (Cai et al. 2010; Cevallos et al. 2017). This would help determine whether improved detection is driven by amplification chemistry or primer binding site selection.

Importantly, this study presents the first field applicable screening tool applied to a non-lethal alternative sample type, gill biopsy. Integrating non-lethal sampling with LAMP for field-based molecular diagnosis is valuable to BFT aquaculture due to the high economic value of the species (Ellis and Kiessling 2016; Carabott et al. 2026a). Current monitoring in BFT relies on lethal sampling, limiting both the number of sampling time points and total number of fish that can be sampled (Carabott et al. 2026b). Non-lethal gill biopsy samples have recently been demonstrated as more sensitive for *C. forsteri* and *C. orientalis* DNA detection than the standard gill necropsy in ranched SBT (Carabott et al. 2026a). Combining LAMP with non-lethal sampling considerably enhances *Cardicola* spp. surveillance by allowing more fish to be monitored by a faster, cost-effective and user-friendly detection method, providing timely actionable data to inform health management decisions in global BFT aquaculture.

Despite these advances in sampling alternatives and field-based DNA amplification, DNA extraction remains a limiting factor for field-based *Cardicola* spp. diagnostics. Currently, DNA extraction requires commercial kits that need a laboratory setting with a centrifuge (Carabott et al. 2023; Power et al. 2023). While LAMP assays are simple enough to be performed in field-based labs, DNA extraction using commercial kits are costly per sample, an important consideration when aiming to increase the number of fish sampled. Unlike PCR, LAMP assays can tolerate less purified or minimally processed DNA for efficient amplification (Poon et al. 2006; Li et al. 2023). Simplified DNA extraction protocols would further improve the accessibility of molecular testing, providing the option to perform diagnosis on board commercial vessels. DNA dipstick is a novel DNA extraction method that takes 30 s to perform (Mason and Botella 2020). The method reduces processing costs where all required components can be made in house and there is no need for specially trained personnel or any equipment, requiring only buffers, a filter paper strip and DNA/RNA free microcentrifuge tubes (Mason and Botella 2020). While the eluted DNA can be stored, direct elution from DNA dipsticks to LAMP has been shown to enable successful LAMP amplification of *Schistosoma japonicum* DNA from mouse and spiked uninfected human samples (Aula et al. 2023). As a result, simplifying DNA extraction would enable the entire *Cardicola* molecular diagnostic workflow to be completed in field-based settings, reducing diagnostic costs and improving efficiency.

While LAMP reliably identifies infected hosts, reporting prevalence alone overlooks the biological consequences of parasite load. Quantifying infection intensity provides a more meaningful measure of disease severity to aid informed health management decisions (Power et al. 2020). LAMP amplification can be monitored in real-time primarily through fluorescence or turbidity, it is not field applicable (Papadakis et al. 2022). Additionally, microfluidic platforms can leverage the LAMP assays and provide the opportunity to detect *Cardicola* species simultaneously. A multiplexed microfluidic LAMP system would provide rapid detection while further reducing sample and reagent consumption, enabling the simultaneous detection of multiple pathogens while combining sample pretreatment, biological separation, biochemical reactions and signal analysis into chips of a few square centimetres (Chen et al. 2023, 2024). However, these systems also require specialised equipment, and while at a lower cost than commercial instruments, elaborate chip design and complex fabrication steps complicates applicability (Chen et al. 2024). Recent advances integrating compact LAMP reaction systems with accessible signal monitoring, including digital colour/pH analysis and paper-based spot counting approaches, have enhanced the capacity for LAMP quantification in limited resource settings (Nguyen et al. 2020; Alvarez et al. 2021; Papadakis et al. 2022; Strachan et al. 2023; Chen et al. 2024). Validating field deployable quantitative LAMP protocols against qPCR could further enhance the utility of LAMP and support timely management decisions in cultured BFT populations.

## Conclusion

This research constitutes the first development and application of species-specific LAMP diagnostics to detect the three *Cardicola* species reported from BFT. Application of LAMP to BFT health monitoring enables rapid prevalence surveys, providing a species-specific, cost-effective and accessible alternative to standard molecular diagnostics of *Cardicola* species. Although qPCR and RPA were more sensitive on synthetic dilutions, LAMP detected more infections from biological samples, highlighting its practical value for field surveillance. With the capability to inform management and improve treatment decisions in BFT aquaculture globally, this study demonstrates that LAMP can be designed to distinguish even closely related parasitic species, providing a rapid and accessible diagnostic approach with potential for field-based application to explore infection dynamics and host parasite-interactions. 

## Supplementary Information

Below is the link to the electronic supplementary material.

Supplementary Material 1 (PDF 2.01 MB)

## Data Availability

No datasets were generated or analysed during the current study.
