# Supplementary material for: Species-specific loop-mediated isothermal amplification (LAMP) assays for the detection of three blood flukes affecting bluefin tuna aquaculture
Source: Parasitol Res. 2026 Sep 30;125(1):135. doi: 10.1007/s00436-026-08775-2 (PMC13710427; doi:10.1007/s00436-026-08775-2)
Supplement: Supplementary file 1 [file 436_2026_8775_MOESM1_ESM.pdf]

Supplementary Table S1. LAMP primers considered for amplification of *Cardicola forsteri* ITS-2 DNA. Primer set NEB P28 were designed using the New England Biolabs LAMP Primer Designer Tool and includes four primer sets (Target amplicon sizes: ID10 = 156 bp; ID40 = 156 bp; ID05 = 156 bp and ID06 = 156 bp). Primer set MC1 (Target amplicon sizes: Set A = 160 bp; Set B = 160 bp; Set C = 160 bp and Set D = 160 bp) and MC2 (Target amplicon sizes: Set A = 131 bp; Set B = 128 bp; Set C = 131 bp; and Set D = 128 bp) were manually designed in Geneious Prime to maximise species specificity. Tick symbol (✓) denotes that the primer is included in the primer set, and cross symbol (×) denotes that the primer is not included.

Name	Sequence (5' → 3')	Primer ID			
**NEB P28**		**10**	**40**	**05**	**06**
F3	CGATATGTGTGTGTTTGTGT	✓	✓	✓	✓
B3	CTAAATTGCAGTCGCAACTT	✓	✓	✓	✓
FIP	GTCACATTAGGAAAGAGCCACATTA GTATTGCATGCATAACATGGAA	✓	✓	✓	✓
BIP	CGCAATCATGTTCGAATTGTGTAT ACCACTATACTAAGCCACGA	✓	✓	✓	✓
LF_V1	CACTGGCGCGCTTGACTAG	✓	×	✓	✓
LF_V2	ACACTGGCGCGCTTGAC	×	✓	×	×
LB_V1	GTAGCGGCTTGTGCTCAGG	✓	✓	×	×
LB_V2	TGTGGTAGCGGCTTGTGC	×	×	✓	×
LB_V3	GTGGTAGCGGCTTGTGCT	×	×	×	✓
**MC1**		**A**	**B**	**C**	**D**
F3	GCGGCTTGGAAATGGTGCTA	✓	✓	✓	✓
B3	GCAGTCGCAACTTGCAAG	✓	✓	✓	✓
FIP_V1	ATGTTATGCATGCAATA GCATACGTGATTGCTTGCT	✓	×	✓	✓
FIP_V2	CTAGTTCCATGTTATGCATGCAATA GCATACGTGATTGCTTGCT	×	✓	×	×
BIP_V1	GCGCGCCAGTGTTAATCA CAATCCCACTCGAATACAC	✓	✓	×	×
BIP_V3	TCAAGCGCGCCAGTGTTAATC CAATCCCACTCGAATACAC	×	×	×	✓
BIP_V4	GCGCGCCAGTGTTAATCA CAATCCCACTCGAATACAC	×	×	✓	×
LF	AACACACACATATCGAGA	✓	✓	✓	✓
LB*	CCTAATGTGACCAGTCGCA	✓	✓	✓	✓
**MC2**		**A**	**B**	**C**	**D**
F3*	ATTTATCGCGACGCCCAA	✓	✓	✓	✓
B3*	GATTGCGACTGGTCACATTA	✓	✓	✓	✓
FIP_V1	ACACACACATATCGAGAA GCGGCTTGGAAATGGTGCTA	✓	×	✓	×
FIP_V2	ACACACACATATCGAGAA GCTTGGAAATGGTGCTA	×	✓	×	✓
BIP	TGTGTATTGCATGCATAACA AAGAGCCACATTATTGA	✓	✓	✓	✓
LF_V1	AAGCAAGCAATCACGTA	✓	✓	×	×
LF_V2	AAGCAATCACGTATGCCAGG	×	×	✓	✓
LB*	CTAGTCAAGCGCGCCAGTG	✓	✓	✓	✓

*Primers not specific to *C. forsteri* ITS-2 DNA, target the same region for *C. opisthorchis* ITS-2 DNA.

Supplementary Table S2. LAMP primers considered for amplification of *Cardicola opisthorchis* ITS-2 DNA. Primer set MC1 (Target amplicon sizes: Set A = 160 bp; Set B = 153 bp; Set C = 160 bp and Set D = 153 bp) and MC2 (Target amplicon sizes: Set A = 128 bp; Set B = 128 bp; Set C = 128 bp; and Set D = 128 bp) were manually designed in Geneious Prime to maximise species specificity. Tick symbol (✓) denotes that the primer is included in the primer set, and cross symbol (×) denotes that the primer is not included.

Name	Sequence (5' → 3')	Primer ID			
**MC1**		**A**	**B**	**C**	**D**
F3	CGGCTTGGAAATGGTGCCA	✓	✓	✓	✓
B3	TGCAGTCGCAACTTGCAGG	✓	✓	✓	✓
FIP_V1	TCCATATTATGCATGCAAA GCATATGTAATGGCTTGTT	✓	✓	✓	✓
BIP_V1	GCGCGCCAGTGTTAATTA CAATCCCACTCGAATACAC	✓	×	×	×
BIP_V2	TCAAGCGCGCCAGTGTTAATT ACTCGAATACACAATTCGA	×	×	✓	×
BIP_V3	TCAAGCGCGCCAGTGTTAATT CAATCCCACTCGAATACAC	×	×	×	✓
BIP_V4	GCGCGCCAGTGTTAATTA ACTCGAATACACAATTCGA	×	✓	×	×
LF	AATGCACACACATTTAGG	✓	✓	✓	✓
LB*	CCTAATGTGACCAGTCGCA	✓	✓	✓	✓
**MC2**		**A**	**B**	**C**	**D**
F3*	ATTTATCGCGACGCCCAA	✓	✓	✓	✓
B3*	GATTGCGACTGGTCACATTA	✓	✓	✓	✓
FIP	ATGCACACACATTTAGGA GCTTGGAAATGGTGCCA	✓	✓	✓	✓
BIP_V1	TTGCATGCATAATATGG AAGAGCCACATTATTAA	✓	✓	×	×
BIP_V2	TTTGCATGCATAATATGG AAGAGCCACATTATTAA	×	×	✓	✓
LF_V1	ACAAGCCATTACATATGCCAG	✓	×	✓	×
LF_V2	AAACAAGCCATTACATA	×	✓	×	✓
LB*	CTAGTCAAGCGCGCCAGTG	✓	✓	✓	✓

*Primers not specific to *C. opisthorchis* ITS-2 DNA, target the same region for *C. forsteri* ITS-2 DNA.

```
82          1
CFOR        ----------------------------------------------------------------------------------
COPIS       actgtgtgattaatgcgaattgcttactgctttgaacatcgacttcttgaacgcatattgcggctacggggtatctcgtggc
CORI        ctgtgtgaattaatgcgaattgcttactgctttgaacatcgacttcttgaacgcatattgcggctacggggtatctcgtggc

.....................................................................................

                                                                 → F3 →                 → F2 →
164         83
CFOR        ---------------------ttttcatttatcgcgacgcccaaaatgtcgcggcttggaaatggtgctacctggcatacgt
COPIS       cacgcctgtccgagggttggcttttcatttatcgcgacgcccaaaatgtcgcggcttggaaatggtgccacctggcatatgt
CORI        cacgcctgtccgagggttggcttttcatttatcgcgacgcccaaaatgtcgtggcttggaaatagtgctacctggcaatagt

.....................................................................................

                            ← LF ←                   ← F1c ←                       → B1c →
246         165
CFOR        gattgcttgcttttctcgatatgtgtgtgtttgtgtgtattgcatgcataacatggaactagtcaagcgcgccagtgttaa
COPIS       aatggcttgttttcctaaatgtgtgtgcatttgtgtgtttgcatgcataatatggaactagtcaagcgcgccagtgttaa
CORI        gattgcttgctattcctagatgtttacgtttgtgtatgtgtgtttgcatacgtagcatggaattagttgagcgcgccagta

.....................................................................................

                                → LB →                       ← B2 ←
328         247
CFOR        tcaataatgtggctctttcctaatgtgaccagtcgcaatcatgttcgaattgtgtattcgagtgggattgtggtagcggctt
COPIS       ttaataatgtggctctttcctaatgtgaccagtcgcaatcatgttcgaattgtgtattcgagtgggattgtggtagcggctt
CORI        ttaattatgtggctctttcctaatgtgaccggtcgcaatcatactcaaattgtgtatttgagtggaattgtggtagcggctt

.....................................................................................

                                                          ← B3 ←
410         329
CFOR        gtgctcaggtcgtggcttagtatagtggttaatcatgcctgagcttgcaagttgcgactgcaatttagtttgaatactccat
COPIS       gtgctcaggtcgtggcttagtatagtggttaatcatgcctgagcctgcaagttgcgactgcaatttagcttaaatactccat
CORI        gtgctcaggttgtggcttagtatagttgttgatcatgcctgaggttgctagttgttactgtaattcggtttaaatgctcaat

.....................................................................................

492         411
CFOR        gtggtttgtcgaatgtgagatgtcgattgatgttttgata------------------------------------------
COPIS       gtggtttgtcgaatgtgaagtgtcgattgatgttttgatac-ttgttgcctaacctcggatcagacgtgaatacccgctgaa
CORI        gtggtttattgagtgtgaaataacgactgacgatgcaatgtttctttgcctaacctcggatcagacgtgaatacccgctgaa

.....................................................................................

524         493
CFOR        --------------------------------
COPIS       cttaagcatatcactaagcggaggaaaag---
CORI        cttaagcatatcactaagcggaggaaaagaaa

.....................................................................................
```

Supplementary Figure S1. ITS-2 sequence alignment of *Cardicola* spp. showing LAMP primer binding regions for the *Cardicola forsteri* (CFOR)-specific assay (MC1 Set A). Multiple sequence alignment was performed using Clustal Omega implemented in Benchling (COPIS = *C. opisthorchis*; CORI = *C. orientalis*). Primer binding sites are indicated according to their position on the target sequence. Primer sequences are shown in the orientation in which they anneal to the target sequence (→ = forward binding site; ← = reverse binding site; grey = mismatched bases relative to *C. forsteri*).

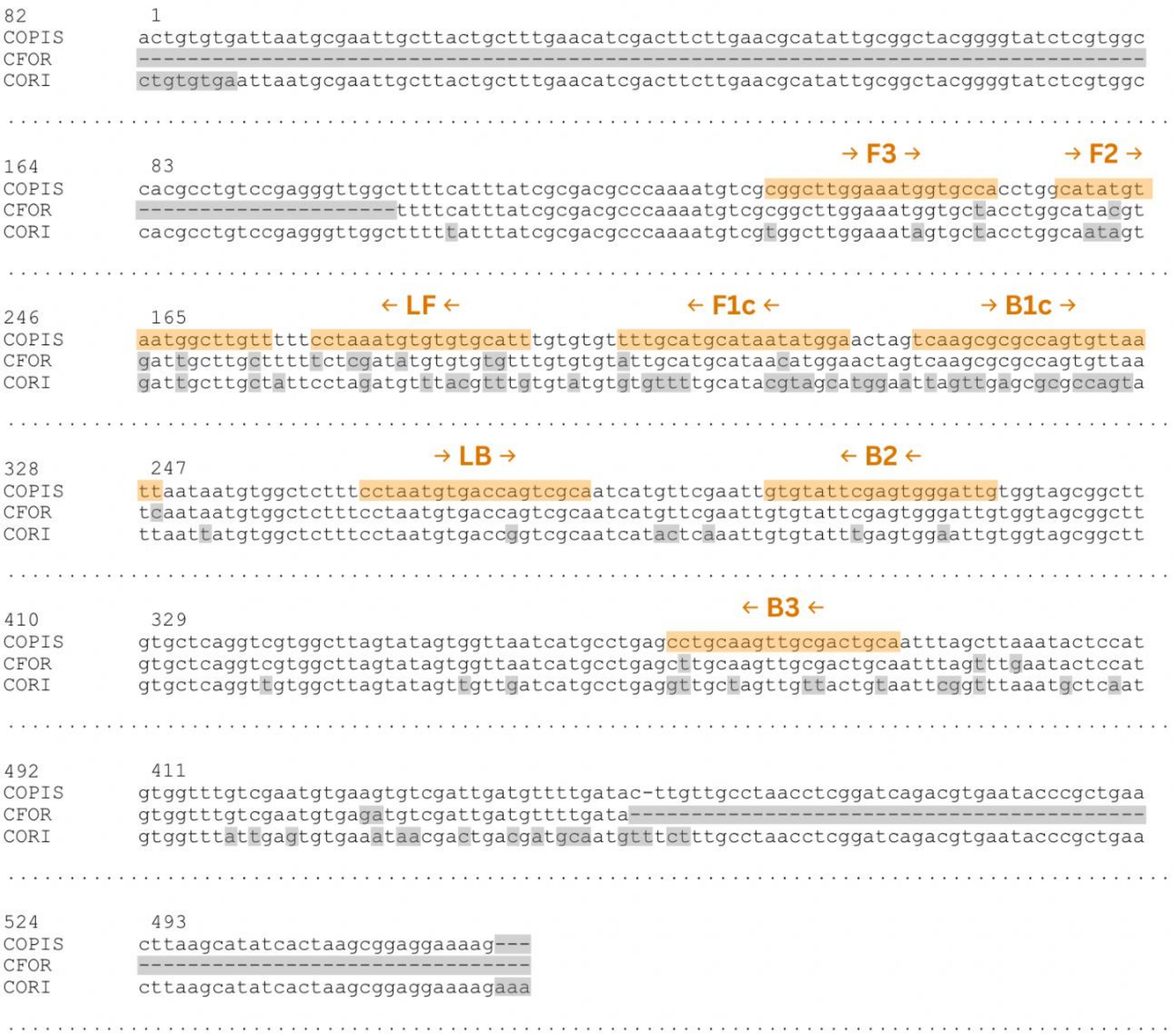

Supplementary Figure S2. ITS-2 sequence alignment of *Cardicola* spp. showing LAMP primer binding regions for the *Cardicola opisthorchis* (COPIS)-specific assay (MC1 Set D). Multiple sequence alignment was performed using Clustal Omega implemented in Benchling (CFOR = *C. forsteri*; CORI = *C. orientalis*). Primer binding sites are indicated according to their position on the target sequence. Primer sequences are shown in the orientation in which they anneal to the target sequence (→ = forward binding site; ← = reverse binding site; grey = mismatched bases relative to *C. opisthorchis*).

```
82            1
CORI          ctgtgtgaattaatgcgaattgcttactgctttgaacatcgacttcttgaacgcatattgcggctacggggtatctcgtggc
CFOR          ----------------------------------------------------------------------------------
COPIS         actgtgtgattaatgcgaattgcttactgctttgaacatcgacttcttgaacgcatattgcggctacggggtatctcgtggc

.....................................................................................

164           83
CORI          cacgcctgtccgagggttggcttttatttatcgcgacgcccaaaatgtcgtggcttggaaatagtgctacctggcaatagt
CFOR          ---------------------ttttcatttatcgcgacgcccaaaatgtcgcggcttggaaatggtgctacctggcatacgt
COPIS         cacgcctgtccgagggttggcttttcatttatcgcgacgcccaaaatgtcgcggcttggaaatggtgccacctggcatatgt

.....................................................................................

                               → F3 →                 → F2 →                 ← LF ←
246           165
CORI          gattgcttgctattcctagatgtttacgtttgtgtatgtgtgttttgcatacgtagcatggaattagttgagcgcgccagta
CFOR          gattgcttgcttttctcgatatgtgtgtgtttgtgtgtattgcatgcataacatggaactagtcaagcgcgccagtgttaa
COPIS         aatggcttgttttcctaaatgtgtgtgcatttgtgtgtttgcatgcataatatggaactagtcaagcgcgccagtgttaa

.....................................................................................

                         ← F1c ←                → B1c →                  → LB →
328           247
CORI          ttaattatgtggctctttcctaatgtgaccggtcgcaatcatactcaaattgtgtatttgagtggaattgtggtagcggctt
CFOR          tcaataatgtggctctttcctaatgtgaccagtcgcaatcatgttcgaattgtgtattcgagtgggattgtggtagcggctt
COPIS         ttaataatgtggctctttcctaatgtgaccagtcgcaatcatgttcgaattgtgtattcgagtgggattgtggtagcggctt

.....................................................................................

                         ← B2 ←                  ← B3 ←
410           329
CORI          gtgctcaggttgtggcttagtatagttgttgatcatgcctgaggttgctagttgttactgtaattcggtttaaatgctcaat
CFOR          gtgctcaggtcgtggcttagtatagtggttaatcatgcctgagcttgcaagttgcgactgcaatttagtttgaatactccat
COPIS         gtgctcaggtcgtggcttagtatagtggttaatcatgcctgagcctgcaagttgcgactgcaatttagcttaaatactccat

.....................................................................................

492           411
CORI          gtggtttattgagtgtgaaataacgactgacgatgcaatgtttctttgcctaacctcggatcagacgtgaatacccgctgaa
CFOR          gtggtttgtcgaatgtgagatgtcgattgatgtttgata-------------------------------------------
COPIS         gtggtttgtcgaatgtgaagtgtcgattgatgtttgatac-ttgttgcctaacctcggatcagacgtgaatacccgctgaa

.....................................................................................

524           493
CORI          cttaagcatatcactaagcggaggaaaagaaa
CFOR          --------------------------------
COPIS         cttaagcatatcactaagcggaggaaaag---

.....................................................................................
```

Supplementary Figure S3. ITS-2 sequence alignment of *Cardicola* spp. showing LAMP primer binding regions for the *Cardicola orientalis* (CORI)-specific assay (NEB P66L39). Multiple sequence alignment was performed using Clustal Omega implemented in Benchling (CFOR = *C. forsteri*; COPIS = *C. opisthorchis*). Primer binding sites are indicated according to their position on the target sequence. Primer sequences are shown in the orientation in which they anneal to the target sequence (→ = forward binding site; ← = reverse binding site; grey = mismatched bases relative to *C. orientalis*).

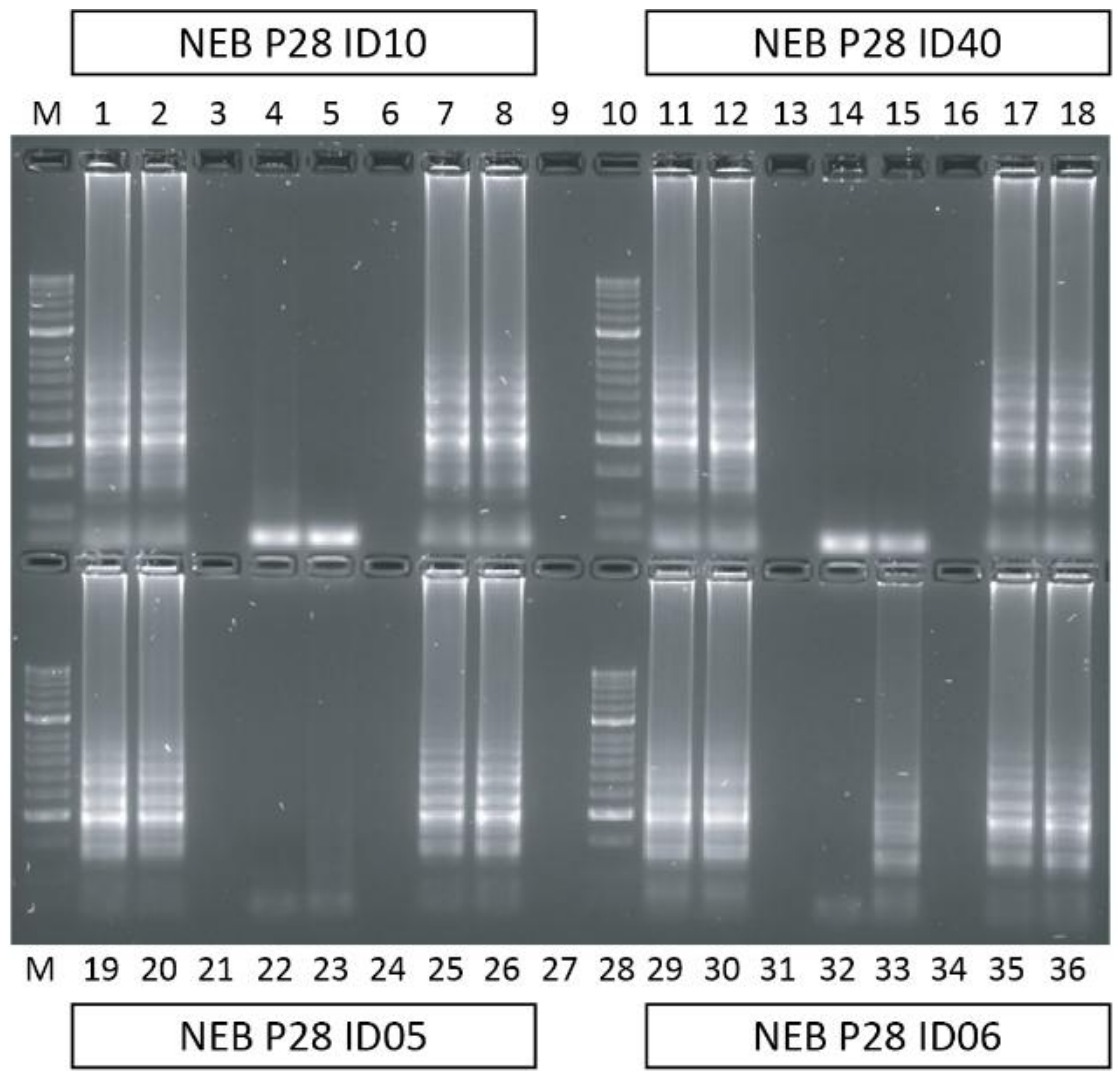

Supplementary Figure S4. Selection of potential LAMP primers for detecting *Cardicola forsteri* ITS-2 DNA designed using the New England Biolabs LAMP Primer Designer Tool. Lane M. 50 bp HyperLadder; Lanes 1-2, 11-12, 19-20, and 29-30. 10 pg/µL *C. forsteri* ITS-2 synthetic DNA; Lanes 4-5, 14-15, 22-23, and 32-33. NTC; Lanes 7-8, 17-18, 25-26, and 35-36. 10 pg/µL *C. opisthorchis* ITS-2 synthetic DNA. All other lanes are empty.

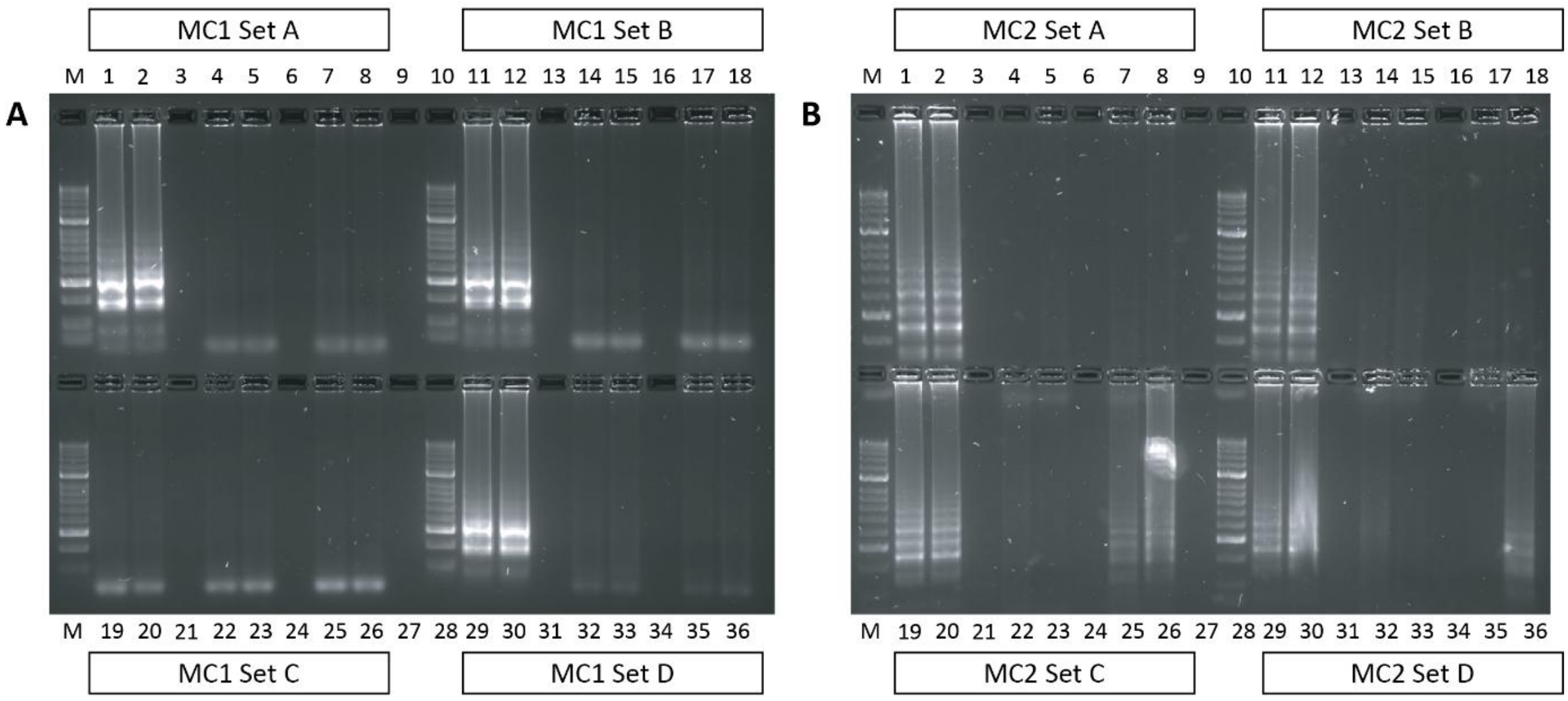

Supplementary Figure S5. Selection of potential LAMP primers for detecting *Cardicola forsteri* ITS-2 DNA manually designed in Geneious Prime. **(A)** Variants of *C. forsteri* MC1 primers. **(B)** Variants of *C. forsteri* MC2 primers. For both panels, Lane M. 50 bp HyperLadder; Lanes 1-2, 11-12, 19-20, and 29-30. 10 pg/µL *C. forsteri* ITS-2 synthetic DNA; Lanes 4-5, 14-15, 22-23, and 32-33. NTC; Lanes 7-8, 17-18, 25-26, and 35-36. 10 pg/µL *C. opisthorchis* ITS-2 synthetic DNA. All other lanes are empty.

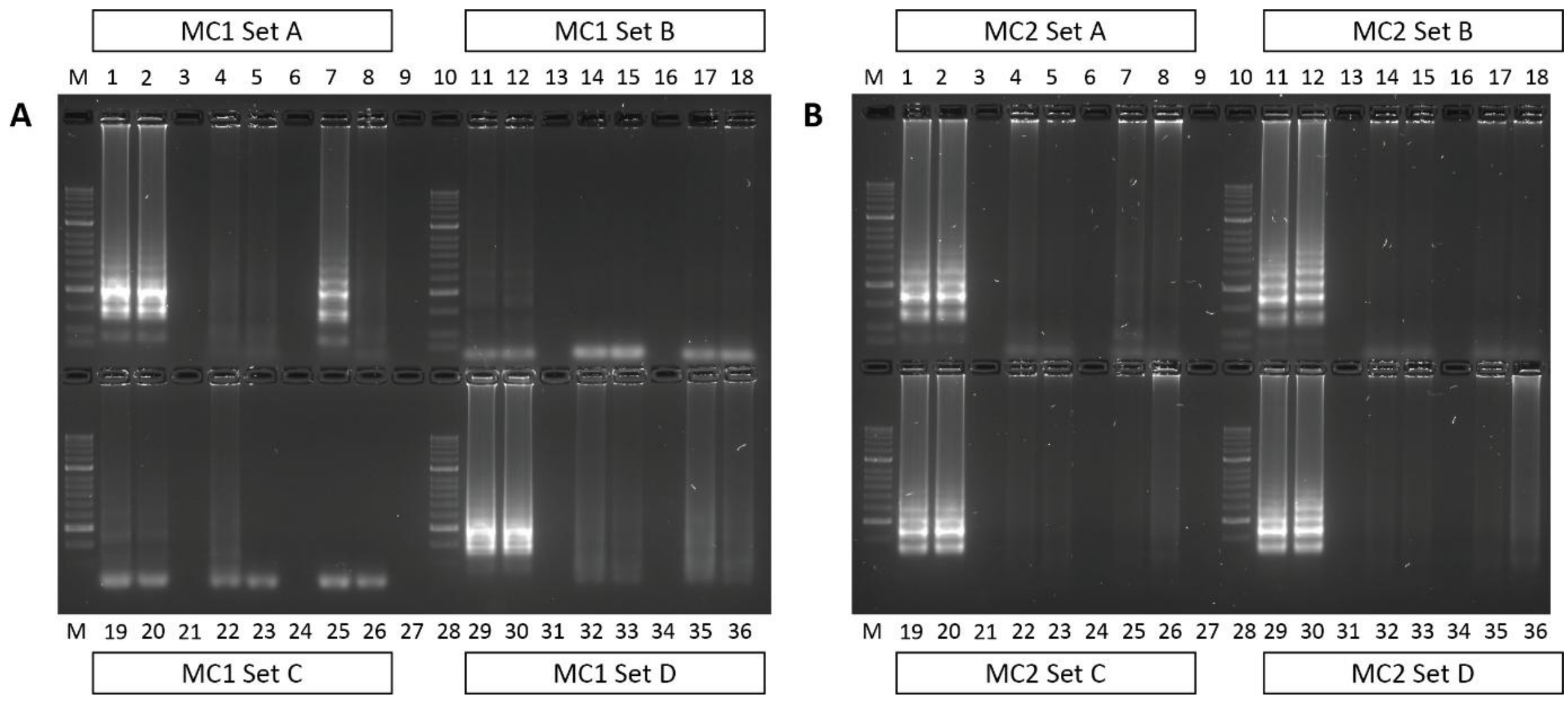

Supplementary Figure S6. Selection of potential LAMP primers for detecting *Cardicola opisthorchis* ITS-2 DNA manually designed in Geneious Prime. **(A)** Variants of *C. opisthorchis* MC1 primers. **(B)** Variants of *C. opisthorchis* MC2 primers. For both panels, Lane M. 50 bp HyperLadder; Lanes 1-2, 11-12, 19-20, and 29-30. 10 pg/µL *C. opisthorchis* ITS-2 synthetic DNA; Lanes 4-5, 14-15, 22-23, and 32-33. NTC; Lanes 7-8, 17-18, 25-26, and 35-36. 10 pg/µL *C. forsteri* ITS-2 synthetic DNA. All other lanes are empty.

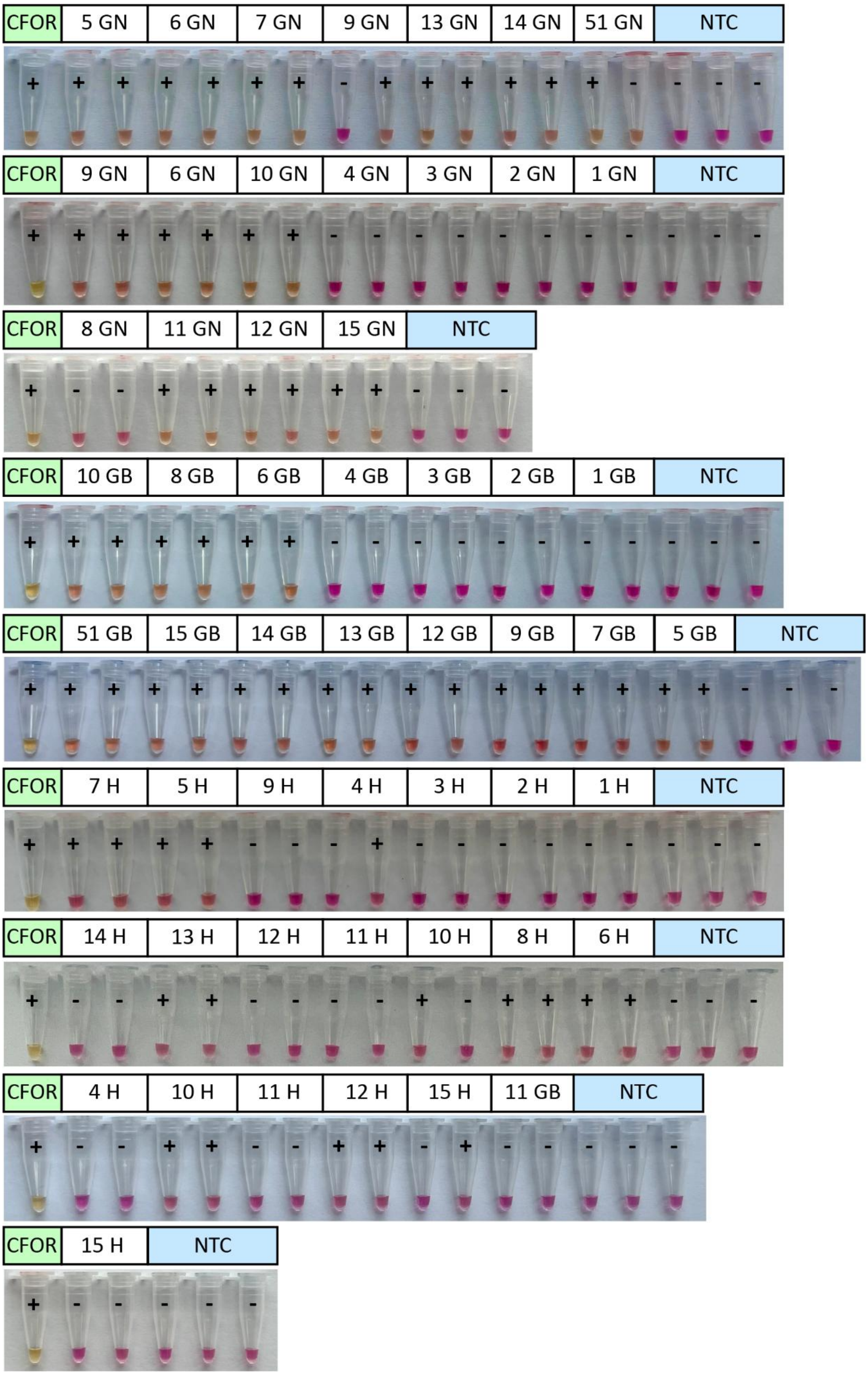

Supplementary Figure S7. Application of LAMP to detect *Cardicola forsteri* (CFOR) ITS-2 DNA from Pacific bluefin tuna heart (H), gill necropsy (GN) and gill biopsy (GB) samples collected from Oshima Station, Aquaculture Research Institute operated by Kindai University, Japan in 2024.

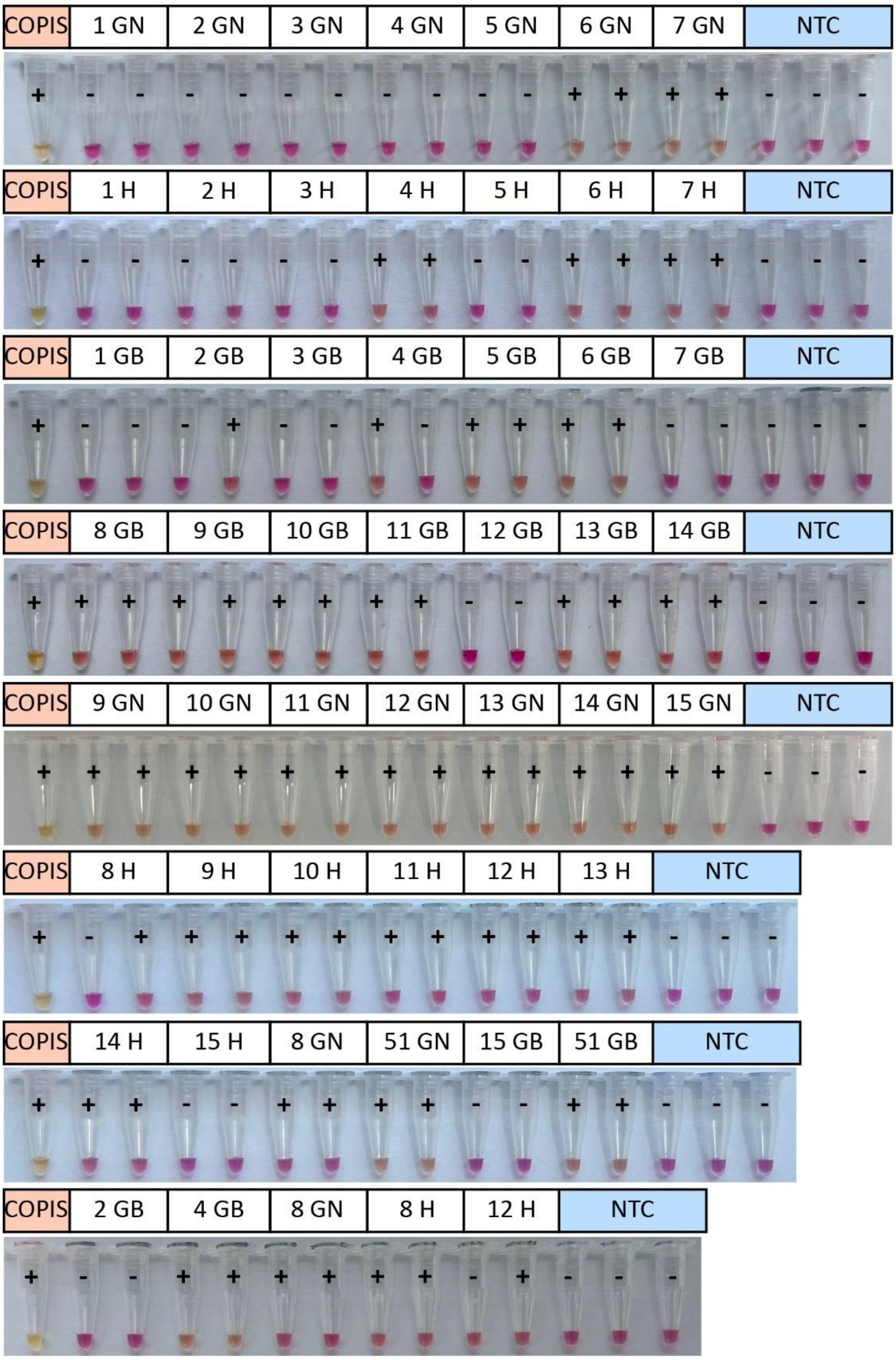

Supplementary Figure S8. Application of LAMP to detect *Cardicola opisthorchis* (COPIS) ITS-2 DNA from Pacific bluefin tuna heart (H), gill necropsy (GN) and gill biopsy (GB) samples collected from Oshima Station, Aquaculture Research Institute operated by Kindai University, Japan in 2024.

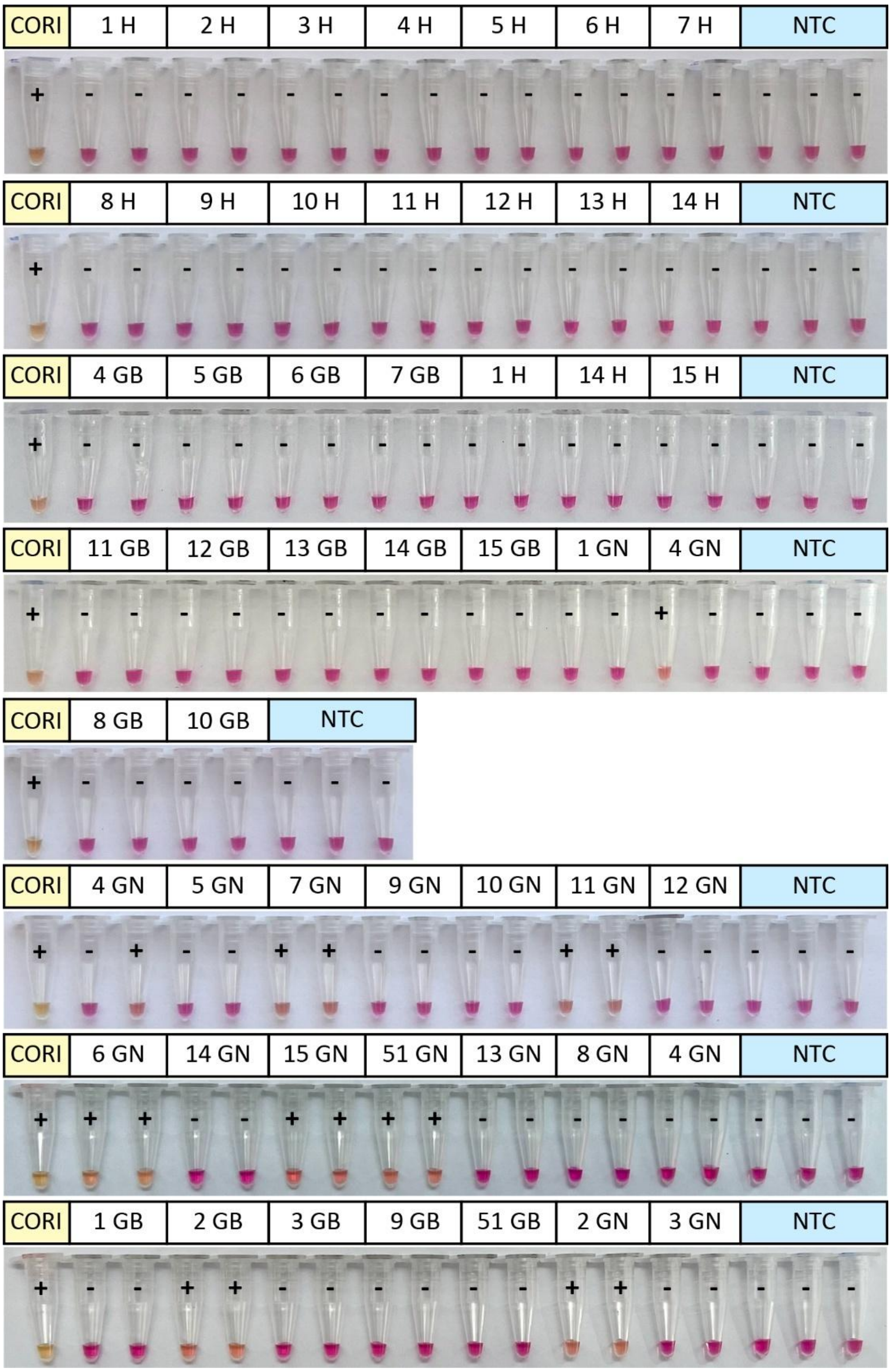

Supplementary Figure S9. Application of LAMP to detect *Cardicola orientalis* (CORI) ITS-2 DNA from Pacific bluefin tuna heart (H), gill necropsy (GN) and gill biopsy (GB) samples collected from Oshima Station, Aquaculture Research Institute operated by Kindai University, Japan in 2024.
